# Advancements in Lignin Valorization for Energy Storage Applications: Sustainable Technologies for Lignin Extraction and Hydrothermal Carbonization

**DOI:** 10.3390/nano15040309

**Published:** 2025-02-18

**Authors:** Haoyu Wang, Haozheng Meng, Joshua O. Olowoyo, Yimin Zeng, Ying Zheng

**Affiliations:** 1 Department of Chemical and Biochemical Engineering, Western University, London, ON N6A 5B9, Canada; hwang928@uwo.ca (H.W.); hmeng43@uwo.ca (H.M.); jolowoyo@uwo.ca (J.O.O.); 2 CanmetMATERIALS, NRCan, Hamilton, ON L8P 0A5, Canada

**Keywords:** lignin, hydrothermal carbonization, lignin extraction technologies, energy storage

## Abstract

The conversion of industrial waste lignin into sustainable carbon materials is an essential step towards reducing dependency on fossil fuels and mitigating environmental impacts. This review explores various aspects of lignin utilization, with particular focus on the extraction of lignin and the application of lignin-derived carbon materials in energy storge applications. The review explores advanced chemical methods to improve the efficiency of biomass conversion, detailing emerging technologies for lignin extraction from various biomasses using innovative solvents and techniques, such as Ionic Liquids and Deep Eutectic Solvents (DESs). Additionally, it discusses the parameters that impact the hydrothermal carbonization (HTC) process. The produced hydrochar shows potential for use as optimized precursors for energy storage applications. This review also considers the implications of these technologies for environmental sustainability and the circular economy, suggesting future research directions to enhance and scale these processes for global impact. This comprehensive analysis highlights the critical role of advanced biomass conversion technologies in achieving sustainability and outlines pathways for future lignin-based carbon materials innovations.

## 1. Introduction

The depletion of fossil fuels and rising environmental concerns have driven significant investigation and innovation aimed at discovering clean and sustainable alternatives. Biowaste, an abundant, renewable resource, has emerged as a promising feedstock to produce energy, fuels, chemicals, and materials in an eco-friendly manner.

Currently, the pulp and paper industry processes vast quantities of lignocellulosic biomass, producing approximately 1.5 × 10^8^–1.8 × 10^8^ tons of technical lignin as an industrial biowaste annually [1,2]. However, less than 2% of the generated lignin waste is converted into high-value products [3,4]. The majority of lignin waste is utilized as low-grade fuel [5]. Lignin is acknowledged as the largest renewable reservoir of aromatic compounds, with an estimated availability of over 3 × 10^11^ tons [6,7]. Lignin contributes to the strength and rigidity of plant cell walls, comprising 15% to 40% *w*/*w* of the dry matter in woody plants and 12% to 21% *w*/*w* in major straw species [8,9]. Given its abundance, lignin can serve as a cost-effective raw material for producing various chemicals and materials, enhancing the economics of lignin-based production lines. As a promising polymer, it is extensively employed for metal ion adsorption and is regarded as the most suitable low-cost adsorbent for purifying trace metals, particularly removing lead ions from water or soil. In addition, lignin finds applications in adhesives, such as in asphalt binders to enhance oxidative aging resistance, in wood adhesive, and as a binder for Li-ion batteries [10,11,12,13,14,15,16,17].

Direct utilization of lignocellulosic or lignin-rich waste presents significant challenges. This is primarily due to its bulkiness, which complicates transportation, handling, and storage. Additionally, they exhibit significantly lower energy density per unit volume or mass compared to fossil fuels [18]. Therefore, converting raw lignin waste into high-value products is essential for practical applications. Biochemical and thermochemical processes are the two main types of established conversion technologies. Biochemical conversion relies on enzymes and microorganisms, primarily involving the fermentation of carbohydrate to produce bioethanol or anaerobic digestion to generate biogas under mild conditions [19,20,21]. However, these processes are highly sensitive to parameters like pH and temperature and are relatively slow, often taking days or weeks to complete, which limits their efficiency [22]. Thermochemical methods, including pyrolysis, gasification, torrefaction, and hydrothermal treatment, demonstrate greater efficiency compared to biochemical conversion, particularly regarding processing time and the ability to convert various types of biowaste feedstocks into liquid and gaseous products (bio-oil and syngas) along with solid products (biochar) [23,24]. However, thermochemical processes generally require higher energy inputs due to elevated temperatures and pressures, which may be offset by their ability to recover energy through high-energy-density products, making the selection of the process context-dependent based on the desired application, feedstock, and energy recovery strategy.

The lignin produced through extraction processes remains in the wet liquor, requiring further treatment to yield a dry lignin product [25,26]. Therefore, hydrothermal carbonization (HTC) presents an optimal method for directly processing this material without the need for drying or centrifugation. Unlike other thermochemical processes, HTC has the distinct advantage of utilizing wet feedstocks without prior dewatering, as water serves as a reactant, solvent, and a catalyst during the process [27,28]. HTC is usually conducted at temperatures between 180 °C and 260 °C, with reaction times ranging from 5 to 240 min or longer and pressures between 10 and 50 bar [2,29]. The hydrochar produced during the HTC process has been extensively used in many applications such as soils amendment, water and gas treatment, energy storage, etc. [2,30].

In recent decades, there has been a rapid increase in studies focused on the valorization of lignin and the hydrothermal carbonization process. Considerable advancements have been achieved in understanding the structure, extraction methods, and potential applications of lignin. This review comprehensively discussed lignin extraction techniques, with particular focus on the studies reported in recent five years. It starts with an overview of the traditional methods, including kraft, sulfite, soda and organosolv processes, and then highlights the newly developed extraction methods. In addition, it examines essential operating parameters and reaction pathways of HTC process for converting lignin into carbon materials, and applications of HTC in the fabrication of lignin-derived carbon materials were deeply explored as well. Additionally, the synthesis routes and potential modification method of various lignin-based carbon materials, and their application in energy storage, were discussed as well.

## 2. Lignin Structure

**Figure 1:**
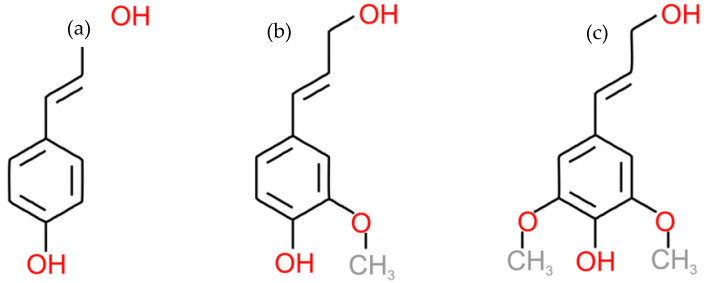
Three common monolignols: (**a**) paracoumaryl alcohol, (**b**) coniferyl alcohol and (**c**) sinapyl alcohol [39].

Lignin represents an amorphous, three-dimensional macromolecular network characterized by heterogeneous structural elements and extended aromatic polymer chains [31]. The molecular weight distributions of lignin exhibit significant variability, attributed to both the inherent diversity of lignin structures across plant species and the influence of extraction methods employed. The number-average molecular weight (M_n_) of lignin ranges from approximately 1000 to 5000 g/mol, while the weight-average molecular weight (M_n_) can range from 2000 to over 50,000 g/mol, which reflects the complex and heterogeneous nature of lignin [32,33,34]. As illustrated schematically below (Figure 1), the primary monomeric components of lignin consist of three phenylpropanoid derivatives: p-hydroxyphenyl (H), guaiacyl (G), and syringyl (S) units, which are derived from their corresponding monolignol precursors: p-coumaryl, coniferyl, and sinapyl alcohols [31,35,36]. These units are distinguished primarily by their aromatic ring methoxylation patterns. Lignin is categorized into three types: softwood lignin comprising approximately 90% of coniferyl alcohol (G), hardwood lignin, containing a mixture of coniferyl (G) and syringyl (S) units, and grass lignin, which includes all three units—G, H and S. The chemical reactivity exhibited by lignin is defined by its extensive variety of oxygen-containing functional groups. Among these functional groups, the hydroxyl and carboxyl groups play particularly significant roles in influencing the properties of the polymer. These functional groups establish a delicate equilibrium between the hydrophilic and hydrophobic characteristics of the lignin macromolecules, while simultaneously regulating their vulnerability to hydrolytic degradation across diverse reaction conditions [37]. The structural integrity of lignin derives from various interunit linkages, including aryl ether bonds (β-O-4, α-O-4, 4-O-5) and carbon–carbon connections (5-5′, β-5, β-1, β-β) [4,36]. Among these, the β-O-4 linkage predominates (approximately 50%), followed by biphenyl (5-5′, 3.5–25%), phenylcoumaran (β-5, 4–10%), and α-aryl ether (α-O-4, 3–5%) bonds. The relatively low bond dissociation energies (BDEs) of these linkages facilitate various chemical and biochemical depolymerization strategies. Within plant cell walls, lignin forms both physical intermolecular interactions and chemical covalent bonds with hemicellulose components [38].

## 3. Lignin Extraction Technologies

The extraction of lignin from lignocellulosic biowastes serves as the initial step in its valorization process. Generally, lignin is bonded to cellulose and hemicellulose via covalent and hydrogen bonds [38]. Thus, a major challenge in the extraction of lignin from lignocellulosic biowaste is the selective and effective breaking of these connections.

### 3.1. Conventional Extraction Technologies

The methodologies used to extract lignin from lignocellulosic waste materials can be broadly categorized into chemical and mechanical approaches, depending on the intended applications of lignin and other residual polysaccharide components. Mechanical processes, mainly referring to grinding, refining and milling, do not represent direct lignin extraction. These technologies promote fiber degradation while preserving lignin inside the biomass matrix. Thus, mechanical pretreatment improves the accessibility and reactivity of the substrate, increasing the effectiveness of subsequent enzymatic and chemical lignin extraction methods [40]. Table 1 summarizes the operating conditions of conventional chemical lignin extraction technologies as well as the chemical and physical properties of the produced lignin.

#### 3.1.1. Kraft Process

Owing to its superior delignification efficiency, the kraft process is extensively employed in pulp and paper manufacturing for lignin extraction. In this process, lignocellulosic biowaste is mixed with NaOH and Na_2_S (white liquor) and heated in a digester at 150–180 °C [47,48]. Under these conditions, lignin undergoes partial depolymerization and is considerably isolated from the carbohydrate matrix by solubilization, producing unbleached kraft pulp and black liquor. The resultant weak black liquor constitutes a complex mixture comprising solubilized kraft lignin, water, various sulfur-containing species, inorganic salts, and additional organic constituents [49]. Additionally, kraft lignin may contain sulfur impurities within its chemical structure due to the use of Na_2_S, which is employed as one of the pulping reagents [50].

Several techniques, including acidification, membrane filtration, and electrolysis, have been developed to isolate lignin from the black liquor. Acidification emerges as the most widely implemented methodology. The acids typically employed for kraft lignin separation from black liquor are H_2_SO_4_, HCl and H_3_PO_4_. H_2_SO_4_ is the most commonly used acid due to its complete dissociation, leading to a more complete precipitation of lignin and ash removal [51,52,53,54,55]. Some researchers have used H_3_PO_4_ as a less corrosive and more effective acidulant [56]. Additionally, H_3_PO_4_ helps to prevent pollution, while H_2_SO_4_ might result in the release of toxic gases [57]. Alternative precipitation agents, including organic acids (acetic, lactic, and citric) have been investigated, offering environmental benefits through the absence of harmful by-products and the potential for CO_2_ capture and utilization [54,58,59,60]. The LignoBoost process represents an innovative technology utilizing CO_2_-induced precipitation through in situ carbonic acid formation. This methodology demonstrates particular efficacy through its ability to integrate with existing kraft pulp facilities, optimizing lignin recovery while maintaining mill chemical cycles [47]. Membrane filtration offers pH and temperature-independent separation alternatives [61,62]. These processes enable selective fractionation based on molecular weight cut-offs, presenting advantages in operational simplicity, energy efficiency, and scalability [63,64]. However, membrane fouling poses significant challenges, necessitating frequent maintenance and potentially increasing operational costs [65]. Electrochemical separation presents an alternative methodology utilizing electrolysis for alkalinity reduction, resulting in anodic lignin electrodeposition and cathodic hydrogen evolution without external chemicals or wastewater production [66,67]. However, the major issues associated with this technology are the high power consumption, the fouling and cleaning of membranes, and the difficulty in removing lignin from the system [68].

#### 3.1.2. Sulfite Process

The sulfite process, recognized as the oldest method for pulping in paper production, facilitates the recovery of lignin as a by-product. It is carried out under acidic or neutral conditions using sulfite salts (SO₃^2−^) or bisulfite salts (HSO_4_^−^) in combination with bases such as Ca^2^⁺, Mg^2^⁺, NH_4_⁺, Na⁺, etc. [69,70]. The key chemical reactions involved in solubilizing lignin during this process include sulfonation, hydrolysis, and condensation [15]. Typically, the sulfite process is conducted at thermal conditions of 125–150 °C which are maintained for 3–7 h, yielding lignosulfonate products [8]. The process involves substantial sulfur incorporation via sulfonation of benzylic carbon positions within the phenylpropane (C9) structural units [71]. The presence of the sulfonic acid groups increases the hydrophilicity, which enhances the water solubility of the modified lignin. Experimental studies have revealed that the typical range of sulfonation lies between 0.4 and 0.7 sulfonic acid groups per phenylpropane unit [72]. Comparative analysis revealed higher sulfur content, carbohydrate residues and inorganic contamination in lignosulfonates relative to kraft lignin [73].

After the sulfite pulping process, lignosulfonates are retained within the spent sulfite liquor, which also contains hemicelluloses and inorganic chemicals, necessitating subsequent extraction for commercial utilization. This separation process involves fermenting residual sugars into ethanol, followed by membrane filtration to reduce inorganic content. Due to the higher molecular weight of lignosulfonates, membrane filtration has been approved as an efficient commercial method for recovering lignosulfonates from spent liquors [47]. However, pressure-driven membrane filtration processes are prone to challenges such as membrane fouling and concentration polarization, which lead to a reduction in membrane flux and hinder production capacity [74]. Commercial usage also includes the Howard process, a four-step procedure that combines washing and precipitation. CaO is first added to the wasted liquor in order to precipitate CaSO_3_, which can be filtered and eliminated. Calcium lignosulfonates, which are solid at pH values higher than 12, are produced with a yield of up to 95% when CaO is added to the solution in the subsequent stage [75].

#### 3.1.3. Soda Process

This method involves alkaline hydrolysis using NaOH, often with anthraquinone, conducted at temperatures up to 165 °C in pressurized reactors to break the ether bonds within the phenolic units of lignin [71]. Similarly to the kraft process, lignin recovery after soda extraction typically involves precipitation as the first step, followed by centrifugation or filtration to isolate the soda lignin from the aqueous phase. However, this may result in co-precipitation of in situ silica with the lignin [76]. The soda process demonstrates a significant advantage in producing sulfur-free lignin fractions, yielding products of enhanced purity compared to kraft lignin [77]. The soda pulping process is particularly suited to extracting lignin from non-wood feedstocks such as grass, straw, bagasse and sugarcane fiber, owing to its moderate severity and compatibility with materials that have lower lignin content [78,79].

#### 3.1.4. Organosolv Process

Organosolv extraction utilizes pure or aqueous organic solvents (ethanol, methanol, phenol, acetone, formic/acetic acids, etc.) with optional catalysts (acids, bases, salts) at temperatures of 130–200 °C [79,80]. The delignification mechanism proceeds through hydrolytic cleavage of α-aryl-ether and lignin–hemicellulose links [80]. This method offers the advantage of extracting low-molecular-weight lignin that is sulfur-free. The lignin produced through this methodology exhibits a high degree of purity attributed to the presence of internal β-O-4 linkages among its molecular units, thereby allowing for the partial retention of its original structure [76]. Additional benefits associated with this technique include a reduced ash content, and the potential for the organic solvent to be reclaimed via distillation processes [81]. Additionally, the properties of this extracted lignin can be enhanced through techniques like distillation or chemical modification to expand its potential applications [82]. Comparative analysis by Gençer et al. of ethanol- and methanol-extracted lignin indicated that the morphological structure and granularity of the lignin produced from these alcohols closely resembled commercial lignin. Additionally, TGA results confirmed superior thermal stability, indicating its potential as an industrial alternative [83].

### 3.2. Emerging Extraction Processes

#### 3.2.1. Deep Eutectic Solvents

Deep eutectic solvents (DESs) represent a specialized class of mixtures exhibiting lower eutectic points relative to their constituent components [84]. They are typically synthesized through the controlled combination of a hydrogen bond donor (HBD) and a hydrogen bond acceptor (HBA) at a specific ratio, resulting in a solvent with distinct physicochemical properties. Due to their low toxicity, biodegradability, and cost-effectiveness, DESs are highly desirable, making them an attractive alternative to traditional solvents for various applications, including the separation and purification of lignin from biomass resources [85]. Lignin dissolves efficiently in DESs due to strong hydrogen bonding between the HBD and the hydroxyl groups in lignin, which disrupts its intermolecular interactions and enhances solubility [86]. HBA also plays a crucial role in tailoring the solvent environment, such as viscosity, acidity, and hydrogen bonding capacity, for optimal lignin dissolution [87,88]. Additionally, halide salt-based HBAs are crucial in enhancing the cleavage of β-O-4 ether bonds through the formation of reactive intermediates, leading to more efficient biomass delignification processes [89]. Among HBAs, choline chloride (ChCl) has been considered as the preferred candidate, offering advantages in terms of environmental sustainability, broad HBD compatibility, and process economics [90]. The HBDs are more varied, with a range of chemicals being commonly utilized [91,92].

Carboxyl acid-based DESs are the solvents most commonly utilized for lignin extraction because of their superior efficiency. Zhang et al. achieved a high delignification rate of 98.5% from corn cob with OA:ChCl [93]. Chen et al. extracted 95% lignin from poplar wood with LA:ChCl at 120 °C over a period of 6 h, yielding 98.1% pure lignin [94]. However, the carboxylic acid-based DES pretreatments require extended treatment periods of 3–24 h [91]. Recently, a microwave-assisted process has been introduced to reduce the processing time. Liu et al. demonstrated that microwave-assisted OA:ChCl DES achieved 80% lignin removal from poplar in just 3 min, matching the performance of conventional heating at 110 °C that required 9 h [95].

Various hydroxyl-containing chemicals are commonly used as HBDs in the synthesis of DESs. Some studies achieved an moderate lignin removal efficiencies of 71.3% and 87.6% from corncob using ChCl–glycerol and ChCl:EG, respectively [93]. Procentese et al. reported comparable findings, indicating that up to 88% of lignin was removed [96]. However, it requires severe pretreatment conditions. Most DESs of this type yield poor results for lignin extraction [90]. The additional hydroxyl groups in HBDs create an extensive hydrogen bond network, increasing viscosity and requiring higher energy input for lignin extraction. This molecular structure also reduces the effectiveness of HBA in disrupting lignin–biomass bonds [97].

Amine and amide compounds (including urea, imidazole, and various amines) have been investigated as HBDs in DES formulations. These types of DESs are generally basic, which promotes the selective dissolution of lignin from biomass via deprotonation of phenolic hydroxyl groups, and exhibit moderate lignin extraction performance [91]. Wheat straw that underwent pretreatment with ChCl–monoethanolamine (ChCl:ME) and ChCl–diethanolamine (ChCl:DE) achieved delignification rates of 81% and 73.5%, respectively [98]. However, ChCl–urea showed lower efficiency (44.74%) even under harsher conditions [99]. This type of DES has similar drawback to previous ones, requiring specific treatment conditions.

DESs that incorporate phenyl groups exhibit potential for biomass delignification through π-π stacking interactions combined with hydrogen bonding with lignin units, leading to disruption of lignin–carbohydrate complexes [90]. Several lignin-derived compounds, including catechol, vanillin, guaiacol, 4-hydroxybenzyl alcohol, *p*-hydroxybenzoic acid (PB), p-coumaric acid (PCA), and 4-hydroxybenzaldehyde (PHA), have been reported as HBDs for synthesizing DESs used in lignin extraction [100,101,102,103]. However, lignin-derived DESs exhibit a lower level of delignification performance. Wang et al. achieved removed 69% of lignin from poplar using ChCl:PB DES [102]. Kim et al. reported comparable results, achieving a maximum lignin removal of 60.8% from switchgrass using ChCl:PCA DES at 160 °C for 3 h [100]. As previously mentioned, DES based on carboxylic acid produced a high lignin yield. Therefore, it is imperative to utilize acidic DESs that will not participate in esterification for the purpose of lignin solubilization. A study conducted by Balasubramanian and Venkatachalam investigated the extraction of lignin from rice husk waste using a DES composed of ChCl and p-toluenesulfonic acid (pTSA). Under optimal conditions, a lignin yield of 87.8% with >97% purity was achieved, demonstrating the DES’s effectiveness under milder conditions compared to other solvent systems. Additionally, the DES formed a stable structure favorable for delignification, as confirmed by theoretical calculations and spectroscopic analysis [104]. However, pTSA-based eutectic solvents exhibit strong acidity, achieving significant removal rates of other lignocellulosic components (e.g., hemicellulose) [105,106]. Thus, ternary eutectic solvents, with reduced pTSA content, can improve performance by balancing acidity and maintaining selectivity [107].

Due to their biodegradability and low toxicity, DESs are considered more environmentally friendly than traditional solvents and align with the principles of green chemistry. The most common method to separate lignin and recover DESs is anti-solvent addition followed by vacuum evaporation, membrane separation techniques, and liquid–liquid extraction. Anti-solvent addition involves adding a solvent like water, acetone or ethanol to precipitate the lignin, then using vacuum evaporation to recover the DES. While straightforward, the efficiency of DES decreases over multiple cycles. Alternatively, membrane separation techniques such as ultrafiltration, microfiltration, and bipolar membrane electrodialysis offer advantages like being solvent-free and having low energy consumption. These approaches can recover 90–98% of the DES components. Liquid–liquid extraction using solvents like 2-methyltetrahydrofuran is more suitable for recovering low molecular weight lignin fractions compared to anti-solvent precipitation [108]. Additionally, DESs have been proven to selectively dissolve lignin in biomass, offering a more targeted method of lignin separation compared to traditional solvents, which may require harsh conditions and result in lower selectivity [109,110]. Compared to conventional methods, the DES approach can achieve higher lignin yields and purities while minimally affecting the fundamental structure of lignin, thereby preserving its natural characteristics [111]. This demonstrates the potential of the DES method to enhance efficiency and product quality. Furthermore, the DES method can provide a faster and more effective process for lignin separation and purification, potentially reducing the time and energy required for the entire process. Additionally, some DESs can be reused over multiple cycles, contributing to the sustainability of the process and potentially reducing overall solvent consumption [91,112]. Overall, the use of DESs for lignin separation and purification holds promise for addressing environmental concerns, improving process efficiency, and enhancing the quality of the extracted lignin, making it an attractive alternative to traditional methods. Table 2 compares the DES method with conventional lignin extraction methods in terms of various aspects.

#### 3.2.2. Ionic Liquid

While deep eutectic solvents (DESs) offer an environmentally friendly approach to lignin extraction, their limitations in solubility and recovery highlight the need for alternative solvents in certain applications. This is where ionic liquids (ILs) come into play. Ionic liquids (ILs) represent low-temperature molten salts (below 100 °C) that are characterized by their unique physicochemical properties, including negligible volatility, thermal robustness, and adaptable solvation characteristics. Unlike DES, ILs are highly versatile and efficient in dissolving both lignin and cellulose, making them a powerful tool for more complex biomass processing. ILs have been explored for various applications, including extracting lignin from biomass [115]. Ionic liquids can modify lignin by reducing its methoxy content and enhancing its reactivity [116]. There are two main methods of extracting lignin using ionic liquids. The first method involves dissolving the entire lignocellulosic biomass (lignin, cellulose, and hemicellulose) in an IL or a mixture of ILs, followed by precipitation of lignin by adding an anti-solvent. The second one is to selectively extract hemicellulose and lignin with ILs, allowing for a more efficient separation of the components. [117] Recent advances focus on protic ionic liquids (PILs), providing particular efficacy in selective lignin recovery [118]. A comprehensive evaluation of 14 PILs conducted by Ghareh et al. identified ethanolammonium acetate ([Eth][Ac]) as optimal, achieving 75.3 ± 3.4% lignin extraction under optimized conditions (95 °C, 20:1 PIL–black liquor ratio, 4.5 h) [115]. FTIR analysis confirmed structural preservation of the extracted lignin, validating PILs as effective extraction media for sustainable lignin recovery. However, the practical application of ILs for lignin extraction faces several challenges. The high cost of ionic liquids, concerns over their toxicity, and issues related to solvent recovery and reuse hinder their widespread industrial adoption [119]. Thus, further research should focus on improved solvent recovery and developing more efficient, sustainable ionic liquids to enable cost-effective lignin valorization.

## 4. Hydrothermal Carbonization of Lignin

Hydrothermal carbonization (HTC) represents a pioneering methodology for converting biomass waste into valuable energy carriers. Compared to other thermochemical conversion processes, HTC operates at significantly lower temperatures and eliminates the need for pre-drying by using water as a solvent. Moreover, gases such as CO_2_, NO_x_ and SO_x_ can dissolve in water, resulting in the formation of corresponding acids and/or salts, which reduces the expenses associated with waste gas treatment processes [120,121,122].

**Figure 2:**
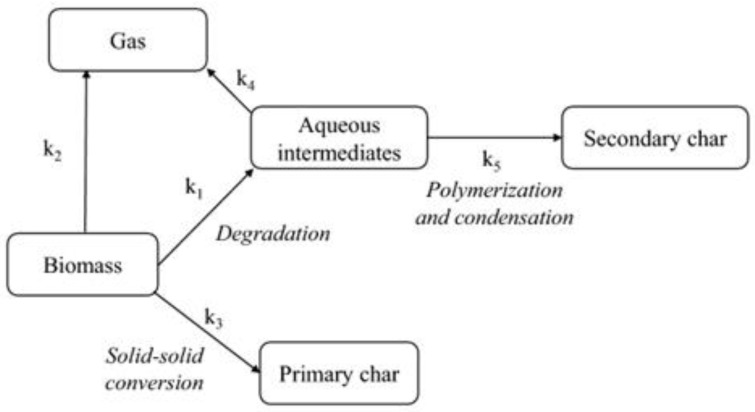
Diagram illustrating the HTC process flow [27].

The HTC process operates in subcritical water conditions, converting wet biomass into a lignite-like biofuel called hydrochar through a series of thermochemical transformations that simulate natural coal formation [115,116,117,118,119]. The HTC process involves multiple reaction mechanisms, including hydrolysis of macromolecules, dehydration of intermediate species, decarboxylation reactions, and subsequent polymerization and aromatization processes. These processes result in partial feedstock dissolution and limited gas formation [123,124]. Figure 2 presents a simplified process flow of HTC. After HTC treatment, the resulting hydrochar features a medium calorific value, high aromatic content, and mesoporous structure. Additionally, thermal processing of hydrochar leads to biological sterilization, and the aqueous by-products can be effectively separated from the hydrochar. This makes it possible to use feedstocks with high moisture content as raw material for hydrochar production [125].

**Figure 3:**
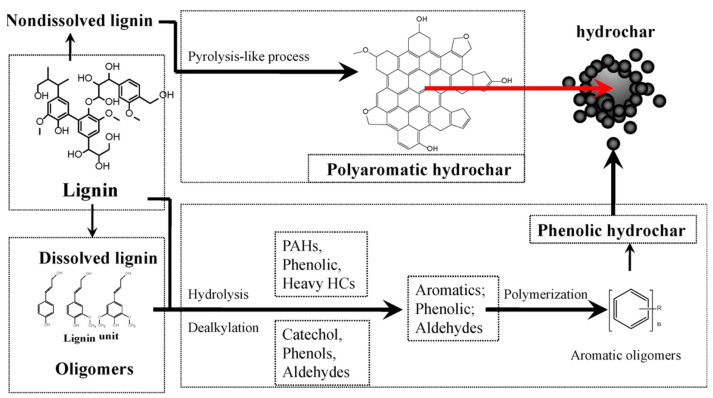
The mechanism and reaction route for hydrochar from lignin [128].

Initially, biowaste undergoes hydrolysis, degrading its complex biomass structure into small molecules [126,127]. This is followed by dehydration, resulting in the removal of water molecules and the formation of intermediates such as furans and aldehydes. These intermediates then undergo polymerization, creating larger molecules and aromatic structures through condensation reactions. As the process continues, further heating and pressurization promote the carbonization of these compounds, leading to the formation of hydrochar, characterized by its high carbon content. During the final stages, decarboxylation and deoxygenation reduce the oxygen content and volatile components in the hydrochar, enhancing its stability and energy content. This carbonization stage plays a critical role in carbon sequestration by generating stable hydrochar structures with reduced oxygen content and volatile matter, thereby offering potential climate mitigation benefits through carbon immobilization. Figure 3 presents the reaction pathways and mechanism for hydrochar derived from lignin. Initially, lignin undergoes hydrolysis and depolymerization, breaking its ether bonds, resulting in phenolic monomers, oligomers, and fragments. This is followed by dehydration and demethoxylation, where hydroxyl and methoxy groups are removed, producing catechols, quinones, and condensed aromatics. Concurrently, decarboxylation and demethylation eliminate carboxyl and methyl groups, releasing CO_2_ and CH_4_ while increasing the aromaticity of the remaining solid structure. As the process progresses, polycondensation promote the formation of larger polycyclic aromatic structures, enhancing the thermal stability and carbon content of hydrochar [128].

Understanding the detailed mechanism of HTC, including the crucial role of physicochemical properties and microstructural changes, enables the optimization of process parameters, such as reaction temperature, residence time, pressure, catalysts, solvent, etc. Enhancing hydrochar quality through the manipulation of reaction conditions can unlock potential applications in energy production, soil amendment, and environmental remediation. Advanced analytical techniques further aid in elucidating changes in functional groups and the structural evolution of hydrochar, offering deeper insights into the process dynamics and efficiency.

Lignin exhibits considerable thermal stability and carbon yield, making it a promising choice for lignin-based carbon materials due to its high aromaticity [129]. HTC can be considered a promising pretreatment method to produce hydrochar, which serves as a superior precursor for generating carbon materials with enhanced porosity [130,131]. Kim et al. successfully extracted lignin from black liquor and transformed it into activated carbon with a highly porous structure [129]. This finding underscores the potential of utilizing industrial waste black liquor as a sustainable source for producing high-quality activated carbon, offering benefits in terms of energy savings and cost-effectiveness. Similarly, Al-kaabi et al. explored and compared two methods—oxidation and HTC—for converting black liquor into hydrochar [132]. They validated that both approaches are effective processes for enhancing and upgrading black liquor into valuable biocarbon with enhanced energy content and reduced ash, which is suitable for use as biofuel and biomaterial. Additionally, the extraction process of lignin significantly impacts the yield of hydrothermal products as well. Latham et al. compared the properties of hydrochar from lignin extracted via the kraft method and the organosolv method [133]. They reported that while the type of lignin does not significantly affect the properties of hydrochar, it does influence the yield. Lignin extracted through the kraft process produces higher hydrochar yields than that obtained via the organosolv method. This difference arises from multiple factors, including the intrinsic properties of the lignin and the specific extraction methodologies used. Kraft lignin, characterized by a predominance of G units with few β-O-4 and α-O-4 linkages, may be more susceptible to hydrothermal conversion, thereby promoting greater hydrochar yield. In contrast, organosolv lignin may exhibit a more intricate array of linkages and structural units, resulting in more lignin fragments dissolving into the liquid phase. The sulfide cooking method employed in kraft lignin production likely induces lignin characteristics favorable for HTC. Additionally, the variation in chemical bonds and interunit connections between kraft and organosolv lignin significantly impacts their decomposition and repolymerization behaviors during hydrothermal treatment. Certain linkages present in kraft lignin may facilitate the formation of more substantial carbon structures and larger hydrochar particles.

### 4.1. Key Parameter of Hydrothermal Carbonization

#### 4.1.1. Reaction Temperature

**Figure 4:**
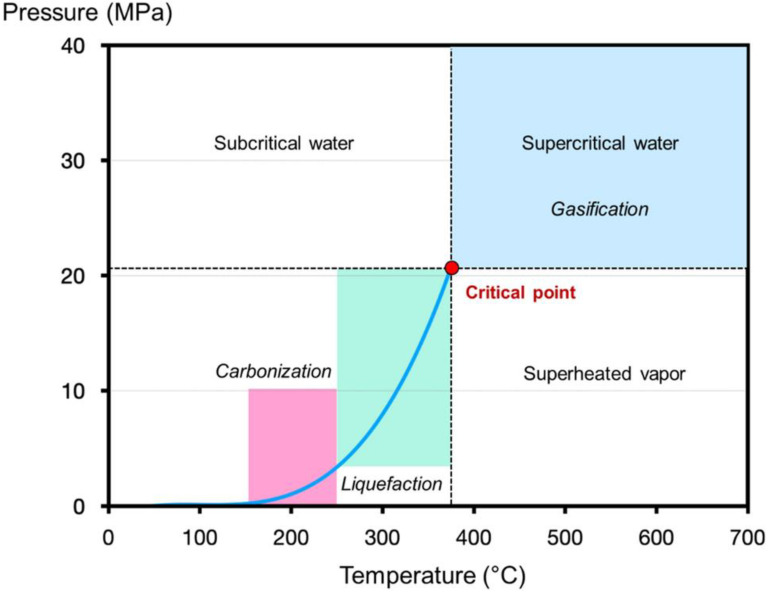
Phase diagram of subcritical and supercritical water [134].

Temperature serves as a critical operational parameter in the HTC process, influencing the properties of water and biomass decomposition kinetics. As shown in Figure 4, HTC is usually conducted in subcritical conditions [2,29]. Under subcritical conditions, water ionization is enhanced, resulting in the generation of hydronium ions (H_3_O^+^) and hydroxide ions (OH^−^), thereby increasing the concentration of H^+^ in subcritical water [134]. In contrast, the reduced dielectric constant characteristic of subcritical water conditions facilitates enhanced solubilization of organic components, enabling the formation of a homogeneous reaction medium that promotes efficient decomposition of biomass-derived fragments [135]. It directly affects the disruption of intermolecular interactions. As temperatures rise, the yield distribution alters, with moderate temperatures promoting oil production and elevated temperatures resulting in enhanced gas formation [121,136]. Solid yields decrease with rising temperatures due to enhanced hydrolysis, dehydration, decarboxylation, and the volatilization of compounds, which also affects the solubilization of feedstock and reduces the carbon content distributed in the solid phase [124,137,138,139,140]. Musa et al. confirmed that increasing HTC temperature from 200 °C to 280 °C results in lignin-derived hydrochar yields progressively reducing from around 88–90 wt.% to 76 wt.%. In addition, the carbon content of the hydrochar increases by 10–15 wt.% as the temperature rises from 200 °C to 280 °C, with a corresponding reduction in oxygen and hydrogen content, leading to a better HHV and energy densification factor. The fuel ratio (fixed carbon/volatile matter) also increases with temperature, indicating greater carbonization [141]. Additionally, the study conducted by Latham et al. found that the chemical structure of the solid hydrochar product of lignin was established in relatively mild HTC conditions, with more severe temperatures mainly decreasing the hydrochar yield rather than altering its chemistry [142]. This wide operating window provides the flexibility to optimize the HTC process for desired products without compromising the solid hydrochar properties.

#### 4.1.2. Reaction Time

Another crucial parameter in HTC process is reaction time. Al-Kaabi et al. [132] report that the mass yield of the hydrochar obtained from black liquor decreases slightly as the reaction time increases. A similar tendency was seen in the investigation conducted by Salcedo-Puerto and co-workers. Increasing the reaction time from 3 to 24 h generally decreased the hydrochar yield from 93% at 3 h to 86% at 24 h due to more extensive degradation. However, the effect on chemical composition and fuel properties is relatively small in the 3–12 h range studied. Very long times (24 h) appear to be detrimental, leading to re-absorption of volatile degradation products and reducing the fuel ratio and thermal stability. The higher heating value is not significantly impacted by reaction time [143]. The removal of ash content is also enhanced as the HTC time increases, which is beneficial for combustion applications [141]. Musa et al. reported that metal removal rates at 6 h were 90%, 92%, 65%, and 58% for Na, K, Ca, and Mg, respectively, compared to 82%, 85%, 57%, and 52% at 1 h. In addition, the solid-state 13C NMR analysis showed that increasing HTC reaction time caused a slight reduction in aliphatic carbon structures and carbonyl groups in the hydrochar. The aromaticity increased from 0.75 to 0.76 as the reaction time was extended from 1 to 6 h, indicating greater cleavage of propyl side chains and methoxy substituents from the aromatic rings at longer times [141]. Overall, reaction time has a more modest influence on hydrochar properties compared to temperature for lignin. The results obtained from multiple studies suggest HTC residence time can be optimized based on throughput and yield considerations without majorly compromising hydrochar quality.

#### 4.1.3. Pressure

The reaction pressure represents a critical process parameter in HTC, influencing both the mechanistic pathways and the resultant physicochemical properties of the produced hydrochar. High pressure is essential in HTC to maintain water in a liquid phase at elevated temperatures, enabling the decomposition and repolymerization of lignin. Most studies employ autogenous pressure conditions. Reactor pressure can be increased through direct temperature elevation or addition of N_2_. According to Le Chatelier’s principle, pressure changes affect reaction equilibrium. At higher pressures, dehydration and decarboxylation reactions are suppressed, while polymerization and condensation reactions are enhanced, leading to the formation of hydrochar [137,144]. Currently, there is a significant knowledge gap regarding the effects of pressure as an independent variable on the hydrothermal carbonization of lignin substrates.

#### 4.1.4. Solvents

The liquid medium in the HTC process serves a dual role, functioning not only as a solvent but also influencing the chemical reactions during the process. Water is the most commonly used solvent in HTC process due to the promotion of hydrolysis, ionic condensation and bond-breaking processes [145]. Al-Kaabi et al. [132] used a mixture of deionized water and acetic acid (99.7%) in a 1:5 ratio as the HTC liquid medium. They compared the products obtained from the HTC of black liquor using this acidic medium to those produced with deionized water alone. Their results revealed that the acidic medium enhanced the removal of alkali metal salts, significantly reducing the ash content in the resulting hydrochar. Compared to using deionized water as the sole medium, incorporating acetic acid substantially lowers the ash content in the hydrochar. Additionally, the hydrochar produced in the acidic medium shows increased carbon, hydrogen, and nitrogen content, along with a higher heating value. However, this enhancement in quality comes with a slight reduction in the yield of solid products. Kang et al. [146] reported that adding formaldehyde to black liquor during HTC significantly improves the yield and quality of hydrochar. Compared to hydrochar produced without formaldehyde, the yield of hydrochar with formaldehyde is 1.27 to 2.13 times higher, and the higher heating value (HHV) is 1.02 to 1.36 times greater. Additionally, the carbon content increases by 1.20 to 2.31 times. The recovery efficiency is also notably enhanced, with total energy recovery efficiency increased by 1.20 to 1.44 times. Furthermore, the sulfur content is reduced by 0.51 to 0.64 times, and the ash content is lowered by 0.48 to 0.89 times.

#### 4.1.5. Catalyst

Catalytic additives can significantly influence the HTC reaction efficiency, products yield, and the properties of the resulting hydrochar. Catalysts can alter reaction pathways, lower the activation energy required for the conversion of biomass to hydrochar, and improve selectivity toward desired products. The use of acid catalysts in HTC significantly enhances the efficiency and quality of hydrochar. Wikberg and co-workers applied H_2_SO_4_ as the catalyst in HTC of kraft lignin. The use of H_2_SO_4_ as a catalyst resulted in a higher hydrochar yield (67%) and carbon recovery (72%) compared to uncatalyzed HTC, with a surface characterized by more spherical particles and pores. In addition, TGA analysis showed that the H_2_SO_4_-catalyzed hydrochar had a lower mass loss (46.9%) compared to the uncatalyzed (53.5%) samples. This indicates that H_2_SO_4_ catalysis produced hydrochar with enhanced thermal stability [147]. Furthermore, Evcil et al. reported that the acid catalysts appear to favor the “soluble pathway” mechanism for hydrochar formation from the lignin components of the biomass. In this pathway, the biomass undergoes hydrolysis into soluble intermediates which then repolymerize into hydrochar, as opposed to direct solid-to-solid conversion. The acid catalysts likely enhance the initial hydrolysis step, enabling the soluble pathway to dominate over the solid-state route [148].

Basic catalysts have demonstrated their ability to enhance the hydrolysis of macromolecules over solid product formation, directing the process toward liquid-phase products. Under alkaline conditions, lignin degrades more readily than cellulose and hemicellulose, producing higher levels of phenolic monomers in the liquid phase [128]. Consequently, alkaline hydrothermal catalysis is unsuitable for hydrochar production, as lignin decomposition reduces the hydrochar yield.

Heterogeneous catalysts can also be employed in the lignin HTC process. These catalysts offer advantages in HTC processing by mitigating corrosion and toxicity issues inherent to homogeneous acid catalyst [149]. However, their successful implementation is hindered by issues of rapid deactivation, operating costs, and environmental impact [149,150]. Liu et al. introduced powdered activated carbon (PAC) in HTC of cotton pulp black liquor (CPBL) [151]. The addition of PAC enhanced pollutant removal efficiency while reducing temperature sensitivity compared to conventional HTC. Additionally, the introduction of PAC promoted the decomposition of lignin macromolecules, with the organic materials adsorbed on the surface of PAC participating in the HTC reaction, achieving in situ catalyst regeneration.

## 5. Lignin-Derived Carbon Materials and Energy Storage Applications

### 5.1. Lignin-Derived Carbon Materials

#### 5.1.1. Porous Carbon

**Figure 5:**
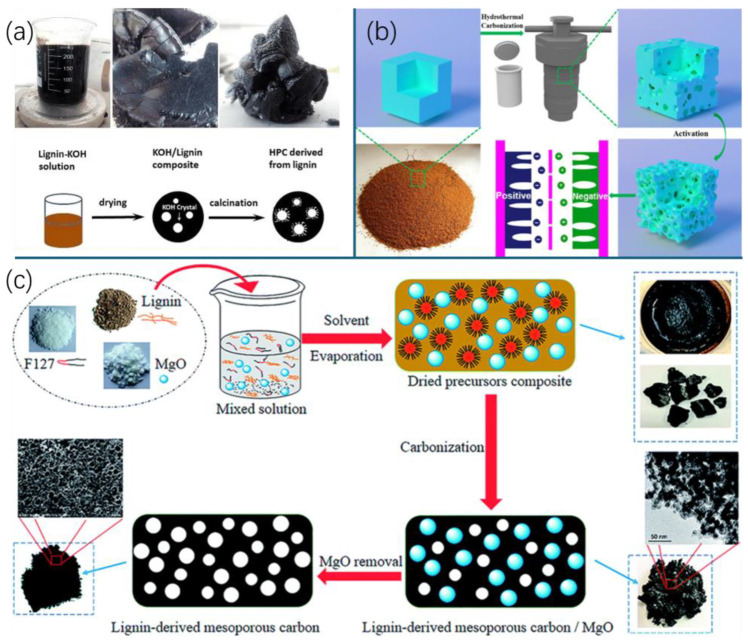
Schematic diagram for conversion approaches to prepare lignin-based porous carbon. (**a**) Direct carbonization with chemical activation [164]; (**b**) hydrothermal carbonization with chemical activation [165]; (**c**) template method [161].

Activated carbon materials with highly developed porosity demonstrate exceptional performance in electrochemical applications, which is attributed to their extensive surface area, enhanced ion adsorption capabilities, superior electrical conductivity, and chemical stability. The inherent three-dimensional framework of lignin provides an advantageous precursor structure for carbonaceous material synthesis [152]. Direct carbonization utilizing activation agents (e.g., KOH, ZnCl_2_, K_2_CO_3_, Na_2_CO_3_, H_3_PO_4_) (Figure 5a) is a highly efficient method to introduce abundant pore structure [153,154]. HTC (Figure 5b) can also be introduced as a promising pretreatment to generate hydrochar as a better precursor for producing a porous carbon material with improved porous characteristics and electrochemical performance [130,131]. Hydrothermal pretreatment creates more oxygen functional groups and thus obtains more active sites for interaction, enhancing the chemical activation process [155,156]. Moreover, the hydrochar produced from HTC contains a macroporous structure, which can be further transferred to porous carbon with a micropore–mesopore–macropore structure via chemical activation at high temperatures [157]. However, corrosive activation agents can lead to significant equipment-related challenges and pose risks to the environment. An alternative approach for acquiring porous carbon from lignin is the template method (Figure 5c) utilizing a hard template (e.g., Mg-, Si-, Zn-, Ca-based materials, etc.) or a soft template (e.g., organic molecules) [153,158,159,160,161]. However, the properties of porous carbons are significantly influenced by the characteristics of hard templates and complex processes to synthesize and remove the templates, whereas the soft template method has limited control over pore structure, resulting in materials with fewer micropores and a smaller specific surface area [162,163].

#### 5.1.2. Cabon Nanofiber (CNF)

**Figure 6:**
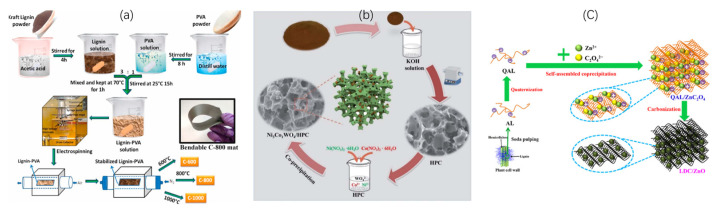
Schematic diagram for conversion approaches to prepare (**a**) lignin-based CNFs with an electrospinning technique [170]; lignin-based carbon composites; (**b**) solvothermal method to prepare Ni_4−x_Co_x_WO_4_/HPC composites [173]; (**c**) carbonization of lignin–metal precursor to prepare LDC/ZnO [174].

Carbon nanofibers (CNFs) are regarded as highly promising materials for electrodes in electrochemical applications, and are recognized for their remarkable electrical conductivity, extensive surface area, elevated aspect ratio, and superior mechanical properties [166,167,168,169]. CNFs can be successfully synthesized from lignin-based polymer composites (PEO, PVA, PNA, etc.) or a solution containing only lignin via a facile electrospinning technique followed by thermal stabilization in an oxidizing atmosphere at 200–280 °C and high-temperature heat treatment under inert atmosphere at 600–1400 °C (Figure 6a) [168,170]. In addition, the pore structure and surface properties of CNFs are able to be modified by applying metal salt additives during the electrospinning process [168]. Currently, there are no studies on using HTC as pretreatment for the production of CNFs. HTC produces carbonaceous materials in powder, hydrochar or amorphous carbon form with a relatively low degree of graphitization [171]. It does not inherently produce the continuous, aligned structures required for carbon fiber formation. However, high degrees of graphitization and alignment are critical for CNF formations [172].

#### 5.1.3. Carbon Composite

An effective way to enhance the electrochemical performance of lignin-derived carbon is introducing metal compounds, metal nanoparticles or conductive polymers to the material to fabricate carbon composites [157,168,173,175,176,177]. One approach involves loading active components onto lignin-derived carbon via a solvothermal method (Figure 6b) [168]. For instance, Shi and the co-workers recently designed a carbon composite containing bimetallic Ni_4−x_Co_x_WO_4_ on lignin-derived 3-D hierarchical porous carbon (HPC) [173]. They mixed Ni(NO_3_)_2_·6H_2_O, Co(NO_3_)_2_·6H_2_O, and the HPC obtained from direct carbonization of enzymatic hydrolysis lignin in deionized water heated at 50 °C for 30 min followed by adding Na_2_WO_4_ and NaOH mixed solution and further heating at 50 °C for 3 h. The obtained solids after centrifugation are the desired Ni_4−x_Co_x_WO_4_/HPC composites. Another approach to producing carbon composites is creating lignin–metal precursor composites before carbonization through the interaction between lignin and metal precursor via coordination, adsorption or chelation (Figure 6c) [168]. Fu et al. successfully developed a composite material composed of lignin-derived carbon and ZnO (LDC/ZnO) [174]. They built a lignin/ZnC_2_O_4_ precursor via electrostatic self-assembly by mixing Na_2_C_2_O_4_ and Zn(NO_3_)_2_·6H_2_O with lignin powder in deionized water and carbonized the obtained precursors at high temperatures after filtration.

#### 5.1.4. Hard Carbon

Hard carbon (HC) exhibits significant potential as an anode material in (SIBs) due to its distinctive electrochemical characteristics, specifically its substantial sodium storage capabilities, pronounced plateau capacity and favorable operating potential. [178]. Lignin serves as a valuable precursor in the synthesis of hard carbon materials, as its abundance of oxygen-containing functional groups enables the formation of porous structures [179]. The electrochemical performance of hard carbon is influenced by several structural characteristics, including graphene interlayer spacing, porosity, particle morphology, surface composition, and crystalline structure [180]. Moreover, the scalability of hard carbon production from lignin presents an intriguing opportunity for sustainable energy solutions, as it not only utilizes a renewable resource but also addresses waste management issues associated with lignin byproducts in various industries [181].

### 5.2. Lignin-Derived Carbon Material for Supercapacitors

Supercapacitors, known for their rapid energy storage capabilities, are widely used in electric vehicles, portable electronics and computers [182]. An optimal carbon electrode must exhibit high electrical conductivity, efficient pore distribution, enhanced wettability, broad availability, cost-effectiveness, and a substantial specific surface area [168,183,184]. The lignin extraction process should ensure minimal cross-linking to promote the development of micropores and mesopores during carbonization. Recent advancements have led to the development of various carbon electrodes derived from lignin specifically designed for enhanced supercapacitor applications.

**Figure 7:**
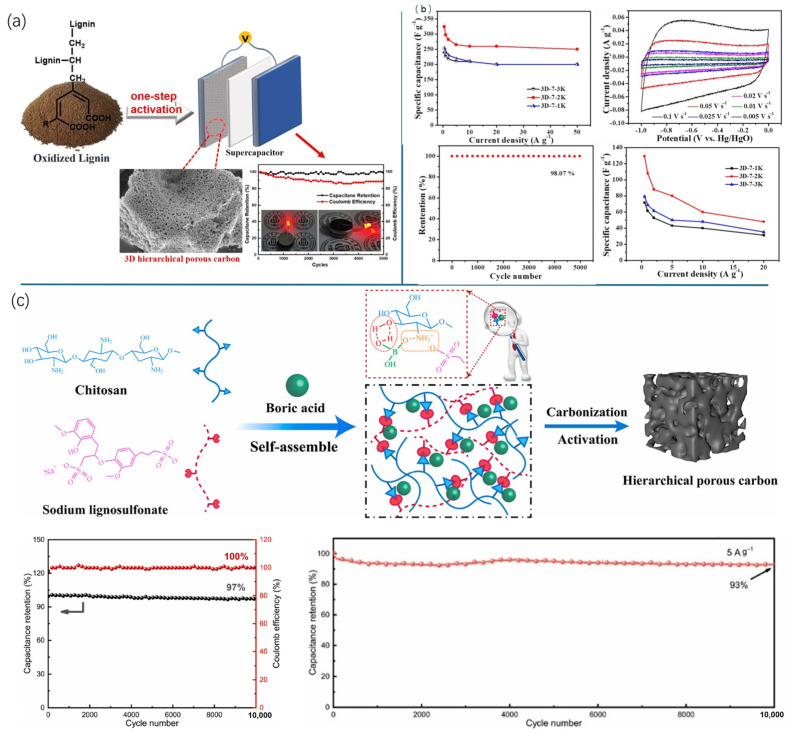
Electrochemical performance of lignin-derived porous carbons in supercapacitors (**a**) hierarchical porous carbon from direct carbonization with KOH activation [185]; (**b**) 3D porous carbon material from HTC followed by KOH activation [165]; (**c**) 3D porous carbon material from soft template method coupled with KOH activation [186].

The development of hierarchical porous carbons from lignin precursors has shown considerable promise for supercapacitor electrode applications, with their three-dimensional structures enabling enhanced electrochemical performance. These materials, prepared through various methods such as direct KOH activation (Figure 7a), hydrothermal carbonization followed by KOH activation (Figure 7b) or a combination of soft templating and KOH activation (Figure 7c), exhibit exceptional electrochemical properties [165,185,186]. With specific surface areas ranging from 1504 to 3094 m^2^/g, these porous carbons achieve specific capacitances of up to 352.9 F/g at current densities of 0.5–1 A/g. In two-electrode configurations, they deliver energy densities of 9.5–28.2 Wh/kg at power densities of 25.0–435.7 W/kg, with some maintaining high energy densities even at ultra-high-power densities. Remarkably, these materials show excellent cycling stability, retaining 88.46–99.7% of their initial capacitance after 5000–10,000 cycles [165,185,186]. These impressive performance metrics can be attributed to the synergistic effects of a high specific surface area, optimized pore size distribution, and an interconnected three-dimensional framework, highlighting the potential of lignin-derived hierarchical porous carbons to advance supercapacitor technology.

Research has demonstrated the effectiveness of CNFs derived from lignin as electrode materials in supercapacitor applications as well, which is attributed to their high specific surface area. Singh et al. synthesized kraft lignin-based CNF using PVA as the polymer binder, achieving a specific capacitance of 196.63 F/g at 1 A/g in a three-electrode system. They constructed symmetrical supercapacitors with aqueous (1 M H_2_SO_4_) and solid-state (PVA-1M H_2_SO_4_ gel) electrolytes. The solid-state device demonstrated a wider potential window (0–2 V), higher power density (1250 W/kg) and energy density (62.65 Wh/kg) at 1 A/g, with 99.5% capacitance retention after 10,000 cycles [170].

**Figure 8:**
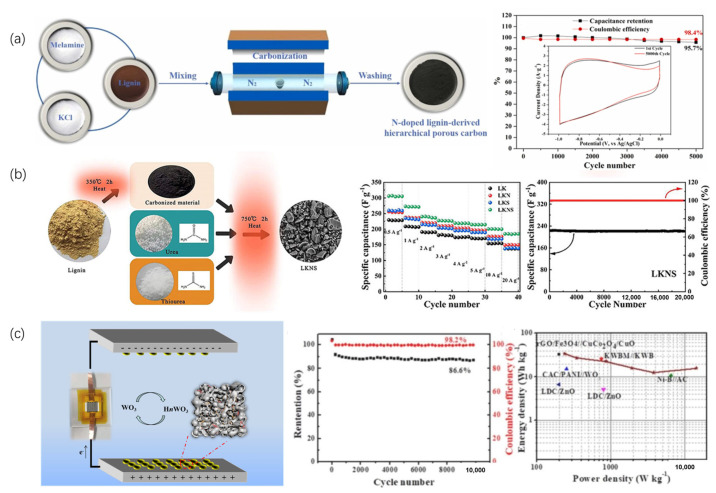
Fabrication of heteroatom-doped lignin-based carbon materials and the electrochemical performance in supercapacitors (**a**) N-doped hierarchical porous carbon [191]; (**b**) N/S co-doped hierarchical porous carbon [193]; (**c**) electrochemical performance of HPC/WO_3_ carbon composite in supercapacitors [177].

Using heteroatom-doping treatment on carbon-based materials to enhance the electrochemical performance of the electrodes for supercapacitors has been widely investigated as well. While many porous carbon materials demonstrate elevated capacitance, their electrical conductivity typically declines as their porosity and specific surface area increase, significantly constraining the power capacity [187]. Incorporating heteroatoms like N, S, P, or B into the carbon framework through direct carbonization of heteroatom-containing precursors or hydrothermal treatment can introduce pseudo-capacitance, improve wettability, and enhance charge transfer capabilities [153,188,189,190,191,192]. Wang et al. (Figure 8a) Wang and colleagues successfully developed N-doped hierarchical porous carbon from lignin precursors, achieving a surface area of 1012.5 m^2^/g and 4.5 at.% N content. This modified material exhibited a specific capacitance of 245 F/g at 0.5 A/g and 98.4% capacitance retention after 5000 cycles, outperforming its nitrogen-free counterpart [191]. Co-doping with multiple heteroatoms can further enhance performance through synergistic effects. Li et al. (Figure 8b) synthesized N/S-doped hierarchical porous carbon using urea and thiourea, achieving a specific capacitance of 232.4 F/g at 0.5 A/g, surpassing single-doped materials. When used as a cathode in zinc ion capacitors, this co-doped carbon exhibited a remarkable specific capacitance of 307 F/g at 1 A/g, outstanding cycling stability with 99.72% capacity retention after 20,000 cycles at 10 A/g, and energy density of 108.8 Wh/kg at 2.88 kW/kg power density [193].

The combination of lignin-derived carbon with metal oxides or conducting polymers enables the synthesis of composite electrode materials that exhibit enhanced electrochemical performance for supercapacitor applications. Shi et al. (Figure 8c) developed a composite material by integrating WO_3_ with lignin-based carbon (HPC/WO_3_). The resulting HPC/WO_3_ composite demonstrated exceptional electrochemical properties. When tested at a current density of 0.5 A/g, it achieved a remarkable specific capacitance of 432 F/g. Long-term stability testing revealed robust performance, with the material retaining 86.6% of its initial capacitance even after 10,000 charge–discharge cycles at a high current density of 10 A/g. In a two-electrode configuration using sulfuric acid electrolyte (1M H_2_SO_4_), the composite delivered an energy density of 34.3 Wh/kg while operating at a power density of 237 W/kg [177].

### 5.3. Lignin-Derived Carbon Material for Anode Materials

#### 5.3.1. Lithium-Ion Batteries

Lithium-ion batteries (LIBs) and sodium-ion batteries (SIBs) are regarded as highly promising technologies for advanced energy storage applications, garnering significant attention in recent years [194]. LIBs have found widespread applications ranging from electronic devices to electric vehicles, due to their superior electrochemical characteristics including high gravimetric and volumetric energy densities coupled with exceptional cycling stability [179,195,196]. While graphite remains the predominant anode material in commercial LIBs [197], its theoretical specific capacity of 372 mAh/g and transport kinetic of Li ion indicate constraints for next-generation energy storage applications demanding high energy and power density [198,199,200]. Moreover, challenges such as overcharging, high current rates and prolonged cycling can cause the delamination of graphite layers to peel and the formation of lithium dendrites, posing a critical safety concern [201]. The specific surface area and porosity of carbon materials are critical for enhancing Li-ion storage capacity. High surface area and mesoporous structures enable fast ion diffusion and provide more active sites for Li-ion storage [202,203]. Extraction methods should prioritize purity and structural control of lignin to reduce interference of Li-ion transport and enhance storage capacity and conductivity. Lignin-derived porous carbon and carbon nanofibers (CNFs) have shown promise as anode materials for LIBs, offering potential improvements over conventional graphite anodes. Lignin-based porous carbon provides hierarchical pore structures and high specific surface areas that facilitate rapid ion transport and large storage sites for Li ions. For example, Xi et al. synthesized a graphite-like porous carbon by activating lignin with K_2_CO_3_, which delivered an initial discharge capacity of 1693 mAh/g at 200 mA/g and maintained 520 mAh/g after 200 cycles, outperforming the theoretical capacity of graphite (372 mAh/g) [196].

In addition to porous carbon, lignin-derived CNFs have gained attention due to their superior electrical conductivity, making them well suited for LIB electrodes. Culebras et al. prepared lignin-based CNFs blended with polylactic acid (PLA) or thermoplastic elastomeric polyurethane (TPU). The lignin/PLA (50:50%) CNFs achieved a specific capacity of 611 mAh/g over a wide potential window (0.011–3 V) after 500 charge/discharge cycles, surpassing graphite’s performance [204].

Despite the promising results, several challenges need to be addressed to fully utilize the potential of lignin-derived carbon materials in high-performance LIBs. One issue is the low initial Coulombic efficiency often observed in these materials. Additionally, there are also sustainability considerations associated with using chemical activation agents and organic polymer blends at high temperatures [180,205,206]. Moreover, lignin-derived carbon materials alone may not meet the energy density criteria for high-performance applications due to their low specific surface areas and insufficient electrical conductivity [202]. The microporous-dominated morphology of lignin-derived carbon materials further restricts ion transport and reduces the overall energy storage capacity [207]. Further research efforts are necessary to address the challenges associated with their practical implementation and to fully unlock their potential in advanced LIB applications.

#### 5.3.2. Sodium-Ion Batteries

**Figure 9:**
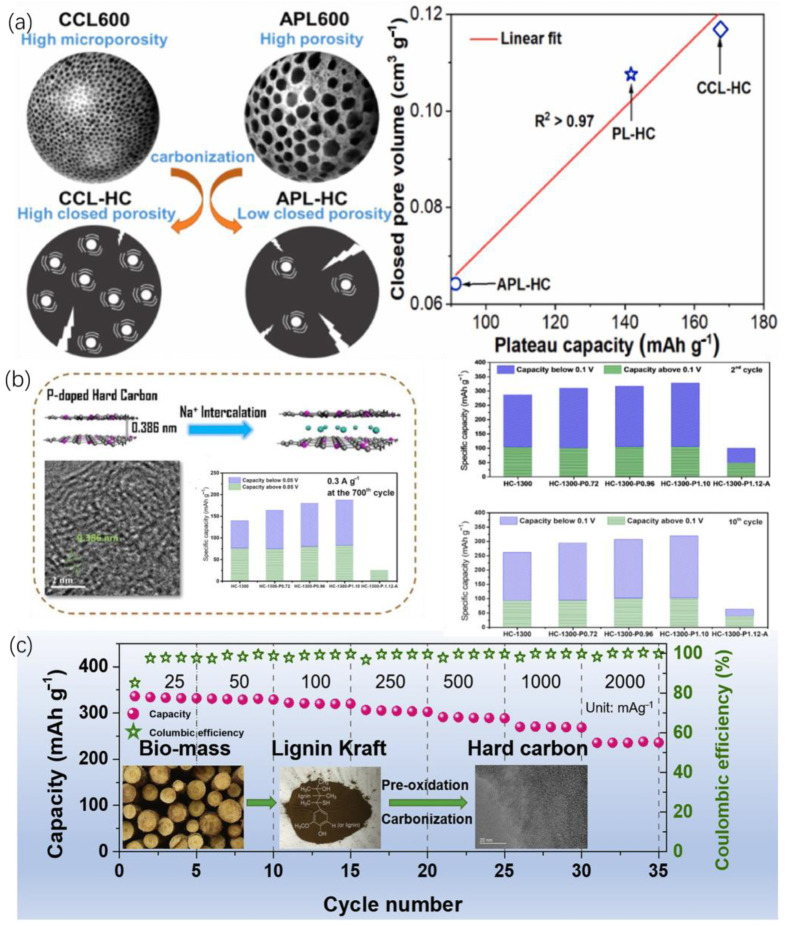
Lignin-derived hard carbons and the electrochemical performance as the anode materials in SIBs (**a**) hard carbon via one-step carbonization [179]; (**b**) P-doped hard carbon [214]; (**c**) hard carbon with pre-oxidation treatment [215].

Due to the limited availability and rising cost of Li, SIBs have gained great interest as a potential alternative for stationary energy storage and electric vehicles, owing to their comparable working principles and chemistry [179,208]. Hard carbon (HC) is regarded as a leading anode material for SIBs due to its high sodium storage capacity, elevated plateau capacity, and low operating voltage [178]. The key structural features include regulated closed pore structure as well as expended interlayer spacing [209,210]. Specific extraction processes that preserve the inherent structural flexibility of lignin are beneficial to prevent excessive densification during carbonization, which can hinder sodium storage capacity. Meng et al. (Figure 9a) produced lignin-based HCs from various precursors, with the highest reversible capacity (292 mAh/g) and initial Coulombic efficiency (62.8%) achieved using corn cob lignin (CCL) at 50 mA/g. The pore structure, influenced by the thermal decomposition of lignin precursors, significantly impacts HC’s electrochemical performance [179]. To further enhance the electrochemical properties and stability of hard carbon anodes, heteroatom doping has been identified as an effective strategy. By introducing heteroatoms into hard carbon, the material undergoes structural changes that improve electrical conductivity, widen interlayer spacing, enhance micropore volume, and produce new defect sites. The addition of heteroatoms to a composite electrode may improve its charge transfer, decrease its diffusion barrier and increase the number of active sites for Na^+^ [211,212,213]. Alvin et al. (Figure 9b) found that P-doped HC exhibited a significantly higher low-voltage plateau capacity (223 mAh/g) and total reversible capacity (328 mAh/g) compared to undoped HC. P-doping also suppressed defects and micropores, maintaining a high initial Coulombic efficiency (72%) and stable performance over 700 cycles at 0.3 A/g [214]. In contrast, other dopants like N and S did not significantly increase the electrochemical performance of hard carbon, likely due to their decomposition at high carbonization temperatures and minimal effect on interlayer spacing. Lin et al. (Figure 9c) proposed a pre-oxidation method to modify the lignin precursor structure, introducing carbonyl groups that enhance crosslinking. This inhibits the development and alignment of graphitic layers during carbonization, increasing interlayer spacing and facilitating sodium-ion insertion. The resulting HC demonstrated exceptional cycling stability, outstanding rate performance and an improved initial Coulombic efficiency (81.4%) [215].

Most studies focus on half-cells; however, analyzing lignin-based anodes within full-cell designs is essential for developing practical batteries. In a full cell, the operating voltage range of the anodes may narrow, especially for sloping voltage profiles. Additionally, because the energy density signal arises from both the cathode and anode, it can be difficult to determine each electrode’s contribution. Lignin-based anodes have demonstrated promising electrochemical cycling stability, often exceeding 100 charge–discharge cycles. However, to ensure their long-term viability in practical applications, it is essential to identify the factors responsible for capacity degradation over extended cycling. Safety considerations such as formation rate and effects of delamination, and the mechanical and thermal stability significantly influence long-term battery performance and must be thoroughly investigated in future research.

## 6. Conclusions and Outlook

Lignin is a valuable resource with a high carbon content and an abundance of functional groups for producing bio-based materials. It has gained significant attention in the fields of supercapacitors and rechargeable batteries owing to its potential to contribute to sustainable development and reduce environmental pollution. This review provides readers with an overview on valorization of lignin. The review focused on various technologies for lignin extraction from lignocellulosic biowaste, including both traditional and emerging techniques. Methods such as kraft, sulfite, soda and organosolv processes, and newer approaches involving DESs and ionic liquids were analyzed. This work also emphasized the importance of HTC process parameters in determining the yield and quality of the resulting hydrochars. These parameters are crucial for optimizing the HTC process. The applications of HTC products as optimized precursors for lignin-based carbon material were explored, demonstrating the potential of HTC technology in resource recovery and environmental protection. In addition, the utilization of different lignin-derived carbon materials in supercapacitors and batteries is discussed. Despite considerable advancements, there are still some challenges which require further research.

### 6.1. Enhancing the Scalability of HTC Processes

HTC has demonstrated significant promise as an efficient technique for transforming high-moisture biomass into carbon-rich fuels at the laboratory scale. However, scaling up to industrial applications presents several challenges, particularly with regard to equipment costs and energy consumption. The high-pressure and high-temperature conditions required for HTC increase both complexity and operational costs, limiting its adoption at an industrial scale. Therefore, future research efforts should focus on developing more energy-efficient and cost-effective reactor designs for HTC. Researchers could explore optimizing reaction conditions to reduce energy consumption while maintaining or improving product quality. Additionally, the integration of heat recovery systems is a promising area of study, as these systems can capture and reuse the heat generated during the HTC process, further reducing energy requirements. Furthermore, the effective utilization of liquid-phase by-products, which often contain valuable organic compounds, should be investigated. These by-products could be processed into valuable chemicals or energy, thereby enhancing the overall economic viability of the HTC process.

### 6.2. Improving the Electrochemical Application of Carbon Materials

Although lignin-derived carbon materials have shown significant potential in electrochemical applications, controlling carbon structure and porosity remains challenging. The properties of the precursor lignin, which are heavily influenced by the extraction process, have significant impact on the morphology, pore structure, surface composition, and ultimately, the battery performance of the resulting carbon material. Furthermore, the variety of modification techniques and carbonization processes introduces additional variability, complicating the optimization of lignin for use as energy storage materials. To address these challenges, future research should focus on obtaining stable and uniform lignin as well as employing advanced operando characterization techniques to elucidate the surface changes and energy storage mechanisms of these materials.

## Figures and Tables

**Table 1:** Traditional lignin extraction technologies and chemical and physical properties of produced lignin [41,42,43,44,45,46].

Process	Condition	S, wt.%	Ash, wt.%	Mw, g/mol	Tg, ℃	Solubility	Purity
Kraft	150–180 °C, NaOH + Na_2_S	1.0–7.0	0.5–3.0	1500–25,000	108–165	Alkali, organic solvents	High
Sulfite	125–150 °C, SO_3_^2−^ or HSO_4_^−^	3.5–8.0	4.0–8.0	1000–50,000	127–154	Water	Low
Soda	Up to 165 °C, NaOH	0	0.7–2.3	1000–15,000	150–155	Alkali	Medium–high
Organosolv	130–200 °C, EtOH	0	~1.7	500–5000	89–97	Organic solvents	Very high

**Table 2:** Comparison of deep eutectic solvents (DESs) and traditional lignin extraction methods based on various parameters [111,113,114].

Parameter	DES	Traditional Methods
**Selectivity and Purity**	High selectivity for lignin.	Lower selectivity for lignin with possible modified structure; often dissolves non-lignin components.
**Environmental Impact**	Biodegradable and low-toxicity solvents, aligned with green chemistry principles.	Often uses toxic chemicals like H2SO4, NaOH, or organic solvents.
**Sustainability and** **Cost-effectiveness**	Relatively cost-effective due to the simplicity of synthesis and potential for reusing over multiple cycles; lignin-derived DESs can create closed-loop systems.	Reusability is limited; traditional solvents may degrade or require regeneration; generates significant chemical waste and extra cost for handling of toxic waste.

## References

[1] Hu J., Zhang Q., Lee D.-J. (2018). Kraft lignin biorefinery: A perspective. Bioresour. Technol..

[2] Cao Y., He M., Dutta S., Luo G., Zhang S., Tsang D.C. (2021). Hydrothermal carbonization and liquefaction for sustainable production of hydrochar and aromatics. Renew. Sustain. Energy Rev..

[3] Nguyen L.T., Phan D.-P., Sarwar A., Tran M.H., Lee O.K., Lee E.Y. (2021). Valorization of industrial lignin to value-added chemicals by chemical depolymerization and biological conversion. Ind. Crops Prod..

[4] Gan M.J., Niu Y.Q., Qu X.J., Zhou C.H. (2022). Lignin to value-added chemicals and advanced materials: Extraction, degradation, and functionalization. Green Chem..

[5] Cao L., Iris K., Liu Y., Ruan X., Tsang D.C., Hunt A.J., Ok Y.S., Song H., Zhang S. (2018). Lignin valorization for the production of renewable chemicals: State-of-the-art review and future prospects. Bioresour. Technol..

[6] Pandian B., Arunachalam R., Easwaramoorthi S., Rao J.R. (2020). Tuning of renewable biomass lignin into high value-added product: Development of light resistant azo-lignin colorant for coating application. J. Clean. Prod..

[7] Shah S., Xu Q., Ullah M., Zahoor S.S., Morales G., Sun J., Zhu D. (2023). Lignin-based additive materials: A review of current status, challenges, and future perspectives. Addit. Manuf..

[8] Torres L.A.Z., Woiciechowski A.L., de Andrade Tanobe V.O., Karp S.G., Lorenci L.C.G., Faulds C., Soccol C.R. (2020). Lignin as a potential source of high-added value compounds: A review. J. Clean. Prod..

[9] Handika S.O., Lubis M.A.R., Sari R.K., Laksana R.P.B., Antov P., Savov V., Gajtanska M., Iswanto A.H. (2021). Enhancing Thermal and Mechanical Properties of Ramie Fiber via Impregnation by Lignin-Based Polyurethane Resin. Materials.

[10] Hou X., Zang Y., Xie L., Liu X., Chen X., Ni S., Zhang F. (2023). Active microlignins derived from formic acid fractionation for adsorption of Pb(II). Mater. Lett..

[11] Du B., Bai Y., Pan Z., Xu J., Wang Q., Wang X., Lv G., Zhou J. (2022). pH fractionated lignin for the preparation of lignin-based magnetic nanoparticles for the removal of methylene blue dye. Sep. Purif. Technol..

[12] Arafat S., Kumar N., Wasiuddin N.M., Owhe E.O., Lynam J.G. (2019). Sustainable lignin to enhance asphalt binder oxidative aging properties and mix properties. J. Clean. Prod..

[13] Hazwan Hussin M., Samad N.A., Latif N.H.A., Rozuli N.A., Yusoff S.B., Gambier F., Brosse N. (2018). Production of oil palm (*Elaeis guineensis*) fronds lignin-derived non-toxic aldehyde for eco-friendly wood adhesive. Int. J. Biol. Macromol..

[14] Theng D., El Mansouri N.E., Arbat G., Ngo B., Delgado-Aguilar M., Pèlach M.A., Fullana-i-Palmer P., Mutjé P. (2017). Fiberboards Made from Corn Stalk Thermomechanical Pulp and Kraft Lignin as a Green Adhesive. Bioresources.

[15] Aro T., Fatehi P. (2017). Production and Application of Lignosulfonates and Sulfonated Lignin. ChemSusChem.

[16] Dieste A., Clavijo L., Torres A.I., Barbe S., Oyarbide I., Bruno L., Cassella F. (2016). Lignin from *Eucalyptus* spp. Kraft Black Liquor as Biofuel. Energy Fuels.

[17] Lu H., Cornell A., Alvarado F., Behm M., Leijonmarck S., Li J., Tomani P., Lindbergh G. (2016). Lignin as a Binder Material for Eco-Friendly Li-Ion Batteries. Materials.

[18] Xu C., Lancaster J. (2008). Conversion of secondary pulp/paper sludge powder to liquid oil products for energy recovery by direct liquefaction in hot-compressed water. Water Res..

[19] Ahorsu R., Medina F., Constantí M. (2018). Significance and challenges of biomass as a suitable feedstock for bioenergy and biochemical production: A review. Energies.

[20] Sawatdeenarunat C., Surendra K., Takara D., Oechsner H., Khanal S.K. (2015). Anaerobic digestion of lignocellulosic biomass: Challenges and opportunities. Bioresour. Technol..

[21] Ward A., Lewis D., Green F. (2014). Anaerobic digestion of algae biomass: A review. Algal Res..

[22] Gollakota A., Kishore N., Gu S. (2018). A review on hydrothermal liquefaction of biomass. Renew. Sustain. Energy Rev..

[23] Seehar T.H., Toor S.S., Sharma K., Nielsen A.H., Pedersen T.H., Rosendahl L.A. (2021). Influence of process conditions on hydrothermal liquefaction of eucalyptus biomass for biocrude production and investigation of the inorganics distribution. Sustain. Energy Fuels.

[24] Azzaz A.A., Khiari B., Jellali S., Ghimbeu C.M., Jeguirim M. (2020). Hydrochars production, characterization and application for wastewater treatment: A review. Renew. Sustain. Energy Rev..

[25] Mazar A., Paleologou M. (2024). Comparison of the effects of three drying methods on lignin properties. Int. J. Biol. Macromol..

[26] Xie M., Chen Z., Xia Y., Lin M., Li J., Lan W., Zhang L., Yue F. (2021). Influence of the lignin extraction methods on the content of tricin in grass lignins. Front. Energy Res..

[27] Ischia G., Fiori L. (2021). Hydrothermal carbonization of organic waste and biomass: A review on process, reactor, and plant modeling. Water Biomass Valorization.

[28] González-Arias J., Sánchez M.E., Cara-Jiménez J., Baena-Moreno F.M., Zhang Z.J.E.C.L. (2022). Hydrothermal carbonization of biomass and waste: A review. Environ. Chem. Lett..

[29] Masoumi S., Borugadda V.B., Nanda S., Dalai A.K. (2021). Hydrochar: A review on its production technologies and applications. Catalysts.

[30] Khan T.A., Saud A.S., Jamari S.S., Ab Rahim M.H., Park J.-W., Kim H.-J. (2019). Hydrothermal carbonization of lignocellulosic biomass for carbon rich material preparation: A review. Biomass Bioenergy.

[31] Ponnusamy V.K., Nguyen D.D., Dharmaraja J., Shobana S., Banu J.R., Saratale R.G., Chang S.W., Kumar G. (2019). A review on lignin structure, pretreatments, fermentation reactions and biorefinery potential. Bioresour. Technol..

[32] Yao H., Wang Y., Liu J., Xu M., Ma P., Ji J., You Z. (2022). Review on applications of lignin in pavement engineering: A recent survey. Front. Mater..

[33] Solihat N.N., Hidayat A.F., Taib M.N.A.M., Hussin M.H., Lee S.H., Ghani M.A.A., Edrus S.S.O.A., Vahabi H., Fatriasari W. (2022). Recent developments in flame-retardant lignin-based biocomposite: Manufacturing, and characterization. J. Polym. Environ..

[34] Tolbert A., Akinosho H., Khunsupat R., Naskar A.K., Ragauskas A.J. (2014). Characterization and analysis of the molecular weight of lignin for biorefining studies. Biofuels Bioprod. Biorefining.

[35] Kanetake T., Sasaki M., Goto M. (2007). Decomposition of a lignin model compound under hydrothermal conditions. Chem. Eng. Technol. Ind. Chem.-Plant Equip.-Process Eng.-Biotechnol..

[36] Zhang C., Wang F. (2020). Catalytic lignin depolymerization to aromatic chemicals. Acc. Chem. Res..

[37] Karmanov A., Ermakova A., Raskosha O., Bashlykova L., Rachkova N., Kocheva L. (2024). Structure and Biomedical Properties of Lignins (A Review). Russ. J. Bioorganic Chem..

[38] Ruiz H.A., Rodríguez-Jasso R.M., Fernandes B.D., Vicente A.A., Teixeira J.A. (2013). Hydrothermal processing, as an alternative for upgrading agriculture residues and marine biomass according to the biorefinery concept: A review. Renew. Sustain. Energy Rev..

[39] Melati R.B., Shimizu F.L., Oliveira G., Pagnocca F.C., de Souza W., Sant’Anna C., Brienzo M. (2019). Key factors affecting the recalcitrance and conversion process of biomass. BioEnergy Res..

[40] Priyadarshi R., Ghosh T., Purohit S.D., Prasannavenkadesan V., Rhim J.-W. (2024). Lignin as a sustainable and functional material for active food packaging applications: A review. J. Clean. Prod..

[41] Gellerstedt G. (2015). Softwood kraft lignin: Raw material for the future. Ind. Crops Prod..

[42] Souza R., Gomes F., Brito E., Costa Lelis R. (2020). A review on lignin sources and uses. J. Appl. Biotechnol. Bioeng..

[43] Bajwa D., Pourhashem G., Ullah A.H., Bajwa S. (2019). A concise review of current lignin production, applications, products and their environmental impact. Ind. Crops Prod..

[44] Ruwoldt J. (2020). A critical review of the physicochemical properties of lignosulfonates: Chemical structure and behavior in aqueous solution, at surfaces and interfaces. Surfaces.

[45] Mukherjee S., Mukhopadhyay S. (2023). Recent progress in sulfur-containing technical lignin-based polymer composites. Express Polym. Lett..

[46] Kazzaz A.E., Fatehi P. (2020). Technical lignin and its potential modification routes: A mini-review. Ind. Crops Prod..

[47] Kienberger M., Maitz S., Pichler T., Demmelmayer P. (2021). Systematic review on isolation processes for technical lignin. Processes.

[48] Li K., Zhong W., Li P., Ren J., Jiang K., Wu W. (2023). Antibacterial mechanism of lignin and lignin-based antimicrobial materials in different fields. Int. J. Biol. Macromol..

[49] Argyropoulos D.D., Crestini C., Dahlstrand C., Furusjö E., Gioia C., Jedvert K., Henriksson G., Hulteberg C., Lawoko M., Pierrou C. (2023). Kraft Lignin: A Valuable, Sustainable Resource, Opportunities and Challenges. ChemSusChem.

[50] Liu J., Li X., Li M., Zheng Y. (2022). Lignin biorefinery: Lignin source, isolation, characterization, and bioconversion. Advances in Bioenergy.

[51] Olgun Ç., Ateş S. (2023). Characterization and Comparison of Some Kraft Lignins Isolated from Different Sources. Forests.

[52] Coura M.R., Demuner A.J., Demuner I.F., Blank D.E., Magalhães Firmino M.J., Borges Gomes F.J., Macedo Ladeira Carvalho A.M., Costa M.M., Henrique dos Santos M. (2023). Technical kraft lignin from coffee parchment. Nord. Pulp Pap. Res. J..

[53] Tarres Q., Aguado R., Dominguez-Robles J., Larraneta E., Delgado-Aguilar M. (2022). Valorization of Kraft Lignin from Black Liquor in the Production of Composite Materials with Poly(caprolactone) and Natural Stone Groundwood Fibers. Polymers.

[54] Sharma M., Simões A., Alves P., Gando-Ferreira L.M. (2022). Efficient Recovery of Lignin and Hemicelluloses from Kraft Black Liquor. KnE Mater. Sci..

[55] Chang G., Wang J., Cui W., Wang C., Zhang J. (2022). Thermal conversion of black liquor solids to monomeric aromatic hydrocarbons based on synergistic catalysis by Na compounds and HZSM-5. Sustain. Energy Fuels.

[56] Sarkar M., Tian C., Jahan M.S. (2018). Activated carbon from potassium hydroxide spent liquor lignin using phosphoric acid. TAPPI J..

[57] Hidayati S., Hadi S., Saputra, Iswanto A.H., Fatriasari W., Solihat N.N., Antov P., Hua L.S. (2023). Characterization of lignin isolated from oil palm empty fruit bunch using phosphoric acid. Biomass Convers. Biorefinery.

[58] Da Silva S.H.F., Gordobil O., Labidi J. (2020). Organic acids as a greener alternative for the precipitation of hardwood kraft lignins from the industrial black liquor. Int. J. Biol. Macromol..

[59] Mardiyati Y., Tarigan E.Y., Prawisudha P., Shoimah S.M., Rizkiansyah R.R., Steven S. (2021). Binderless, All-Lignin Briquette from Black Liquor Waste: Isolation, Purification, and Characterization. Molecules.

[60] Mota I.F., da Silva Burgal J., Antunes F., Pintado M.E., Costa P.S. (2023). High value-added lignin extracts from sugarcane by-products. Int. J. Biol. Macromol..

[61] Jönsson A.-S., Nordin A.-K., Wallberg O. (2008). Concentration and purification of lignin in hardwood kraft pulping liquor by ultrafiltration and nanofiltration. Chem. Eng. Res. Des..

[62] Kevlich N.S., Shofner M.L., Nair S. (2017). Membranes for Kraft black liquor concentration and chemical recovery: Current progress, challenges, and opportunities. Sep. Sci. Technol..

[63] Pola L., Collado S., Oulego P., Díaz M. (2022). Kraft black liquor as a renewable source of value-added chemicals. Chem. Eng. J..

[64] He Y., Bagley D.M., Leung K.T., Liss S.N., Liao B.-Q. (2012). Recent advances in membrane technologies for biorefining and bioenergy production. Biotechnol. Adv..

[65] Sharma M., Alves P., Gil M.H., Gando-Ferreira L.M. (2022). Fractionation of black liquor using ZnO nanoparticles/PES ultrafiltration membranes: Effect of operating variables. J. Clean. Prod..

[66] Oliveira R., Mateus M., Santos D. (2018). Chronoamperometric and chronopotentiometric investigation of Kraft black liquor. Int. J. Hydrogen Energy.

[67] Nong G., Zhou Z., Wang S. (2015). Generation of Hydrogen, Lignin and Sodium Hydroxide from Pulping Black Liquor by Electrolysis. Energies.

[68] Fatehi P., Chen J., Fang Z., Smith J.R.L. (2016). Extraction of Technical Lignins from Pulping Spent Liquors, Challenges and Opportunities. Production of Biofuels and Chemicals from Lignin.

[69] Haq I., Mazumder P., Kalamdhad A.S. (2020). Recent advances in removal of lignin from paper industry wastewater and its industrial applications–A review. Bioresour. Technol..

[70] Calvo-Flores F.G., Dobado J.A., Isac-García J., Martín-Martínez F.J. (2015). Lignin and Lignans as Renewable Raw Materials: Chemistry, Technology and Applications.

[71] Tribot A., Amer G., Alio M.A., de Baynast H., Delattre C., Pons A., Mathias J.-D., Callois J.-M., Vial C., Michaud P. (2019). Wood-lignin: Supply, extraction processes and use as bio-based material. Eur. Polym. J..

[72] Brudin S., Schoenmakers P. (2010). Analytical methodology for sulfonated lignins. J. Sep. Sci..

[73] Vishtal A., Kraslawski A. (2011). Challenges in industrial applications of technical lignins. BioResources.

[74] Humpert D., Ebrahimi M., Czermak P. (2016). Membrane technology for the recovery of lignin: A review. Membranes.

[75] Cheng K. (2021). Industrial Scale Lignin Recovery from Pulping Liquors.

[76] Lobato-Peralta D.R., Duque-Brito E., Villafan-Vidales H.I., Longoria A., Sebastian P., Cuentas-Gallegos A.K., Arancibia-Bulnes C.A., Okoye P.U. (2021). A review on trends in lignin extraction and valorization of lignocellulosic biomass for energy applications. J. Clean. Prod..

[77] Fernández-Rodríguez J., Erdocia X., Hernández-Ramos F., Gordobil O., Alriols M.G., Labidi J. (2020). Direct lignin depolymerization process from sulfur-free black liquors. Fuel Process. Technol..

[78] Klett A.S. (2017). Purification, Fractionation, and Characterization of Lignin from Kraft Black Liquor for Use as a Renewable Biomaterial. Ph.D. Thesis.

[79] Ariyanta H.A., Santoso E.B., Suryanegara L., Arung E.T., Kusuma I.W., Taib M.N.A.M., Hussin M.H., Yanuar Y., Batubara I., Fatriasari W. (2023). Recent progress on the development of lignin as future ingredient biobased cosmetics. Sustain. Chem. Pharm..

[80] Rodríguez A., Espinosa E., Domínguez-Robles J., Sánchez R., Bascón I., Rosal A.J.P., Kazi S.N. (2018). Different solvents for organosolv pulping. Pulp and Paper Processing.

[81] Doherty W.O., Mousavioun P., Fellows C.M. (2011). Value-adding to cellulosic ethanol: Lignin polymers. Ind. Crops Prod..

[82] Hidayati S., Fonny Budiyanto E., Saputra H., Hadi S., Heri Iswanto A., Nurfajrin Solihat N., Antov P., Seng Hua L., Fatriasari W., Sapuan Salit M. (2023). Characterization of Formacell Lignin Derived from Black Liquor as a Potential Green Additive for Advanced Biocomposites. J. Renew. Mater..

[83] Gençer A., Akyüz M., Yurdakurban F., Aydemir D. (2022). Characterization and Separation of Lignin from Kraft Black Liquor with Different Alcohols. Drv. Ind..

[84] Martins M.A., Pinho S.P., Coutinho J.A. (2019). Insights into the nature of eutectic and deep eutectic mixtures. J. Solut. Chem..

[85] Liu Z., Hou Y., Liu C., Hu S. (2023). Efficient and eco-friendly isolation and purification of lignin from black liquor with choline chloride-based deep eutectic solvents. Nord. Pulp Pap. Res. J..

[86] Oh Y., Park S., Jung D., Oh K.K., Lee S.H. (2020). Effect of hydrogen bond donor on the choline chloride-based deep eutectic solvent-mediated extraction of lignin from pine wood. Int. J. Biol. Macromol..

[87] Farooq M.Q., Abbasi N.M., Smith E.A., Petrich J.W., Anderson J.L. (2022). Characterizing the solvation characteristics of deep eutectic solvents composed of active pharmaceutical ingredients as a hydrogen bond donor and/or acceptor. ACS Sustain. Chem. Eng..

[88] Li W.-X., Xiao W.-Z., Yang Y.-Q., Wang Q., Chen X., Xiao L.-P., Sun R.-C. (2021). Insights into bamboo delignification with acidic deep eutectic solvents pretreatment for enhanced lignin fractionation and valorization. Ind. Crops Prod..

[89] Da Costa Lopes A.M., Gomes J.R., Coutinho J.A., Silvestre A.J. (2020). Novel insights into biomass delignification with acidic deep eutectic solvents: A mechanistic study of β-O-4 ether bond cleavage and the role of the halide counterion in the catalytic performance. Green Chem..

[90] Zhou M., Fakayode O.A., Yagoub A.E.A., Ji Q., Zhou C. (2022). Lignin fractionation from lignocellulosic biomass using deep eutectic solvents and its valorization. Renew. Sustain. Energy Rev..

[91] Chen Z., Ragauskas A., Wan C. (2020). Lignin extraction and upgrading using deep eutectic solvents. Ind. Crops Prod..

[92] Wang W., Lee D.-J. (2021). Lignocellulosic biomass pretreatment by deep eutectic solvents on lignin extraction and saccharification enhancement: A review. Bioresour. Technol..

[93] Zhang C.-W., Xia S.-Q., Ma P.-S. (2016). Facile pretreatment of lignocellulosic biomass using deep eutectic solvents. Bioresour. Technol..

[94] Chen Y., Zhang L., Yu J., Lu Y., Jiang B., Fan Y., Wang Z. (2019). High-purity lignin isolated from poplar wood meal through dissolving treatment with deep eutectic solvents. R. Soc. Open Sci..

[95] Liu Y., Chen W., Xia Q., Guo B., Wang Q., Liu S., Liu Y., Li J., Yu H. (2017). Efficient cleavage of lignin–carbohydrate complexes and ultrafast extraction of lignin oligomers from wood biomass by microwave-assisted treatment with deep eutectic solvent. ChemSusChem.

[96] Procentese A., Johnson E., Orr V., Campanile A.G., Wood J.A., Marzocchella A., Rehmann L. (2015). Deep eutectic solvent pretreatment and subsequent saccharification of corncob. Bioresour. Technol..

[97] Xia Q., Liu Y., Meng J., Cheng W., Chen W., Liu S., Liu Y., Li J., Yu H. (2018). Multiple hydrogen bond coordination in three-constituent deep eutectic solvents enhances lignin fractionation from biomass. Green Chem..

[98] Zhao Z., Chen X., Ali M.F., Abdeltawab A.A., Yakout S.M., Yu G. (2018). Pretreatment of wheat straw using basic ethanolamine-based deep eutectic solvents for improving enzymatic hydrolysis. Bioresour. Technol..

[99] Pan M., Zhao G., Ding C., Wu B., Lian Z., Lian H. (2017). Physicochemical transformation of rice straw after pretreatment with a deep eutectic solvent of choline chloride/urea. Carbohydr. Polym..

[100] Kim K.H., Dutta T., Sun J., Simmons B., Singh S. (2018). Biomass pretreatment using deep eutectic solvents from lignin derived phenols. Green Chem..

[101] Chen L., Yu Q., Wang Q., Wang W., Qi W., Zhuang X., Wang Z., Yuan Z. (2019). A novel deep eutectic solvent from lignin-derived acids for improving the enzymatic digestibility of herbal residues from cellulose. Cellulose.

[102] Wang Y., Meng X., Jeong K., Li S., Leem G., Kim K.H., Pu Y., Ragauskas A.J., Yoo C.G. (2020). Investigation of a lignin-based deep eutectic solvent using p-hydroxybenzoic acid for efficient woody biomass conversion. ACS Sustain. Chem. Eng..

[103] Huang C., Zhan Y., Cheng J., Wang J., Meng X., Zhou X., Fang G., Ragauskas A.J. (2021). Facilitating enzymatic hydrolysis with a novel guaiacol-based deep eutectic solvent pretreatment. Bioresour. Technol..

[104] Balasubramanian S., Venkatachalam P. (2023). Valorization of rice husk agricultural waste through lignin extraction using acidic deep eutectic solvent. Biomass Bioenergy.

[105] Wu W., Zhu P., Luo L., Lin H., Tao Y., Ruan L., Wang L., Qing Q. (2024). p-Toluenesulfonic acid enhanced neutral deep eutectic solvent pretreatment of soybean straw for efficient lignin removal and enzymatic hydrolysis. Bioresour. Technol..

[106] Gómez-Cruz I., Seixas N., Labidi J., Castro E., Silvestre A.J., da Costa Lopes A.M. (2024). Delignification of Olive Tree Pruning Using a Ternary Eutectic Solvent for Enhanced Saccharification and Isolation of a Unique Lignin Fraction. ACS Sustain. Chem. Eng..

[107] Poy H., Lladosa E., Arcís A., Gabaldón C., Loras S. (2023). Microwave-assisted ternary deep eutectic solvent pretreatment for improved rice straw saccharification under mild pretreatment conditions. Ind. Crops Prod..

[108] Lobato-Rodríguez Á., Gullón B., Romaní A., Ferreira-Santos P., Garrote G., Del-Río P.G. (2023). Recent advances in biorefineries based on lignin extraction using deep eutectic solvents: A review. Bioresour. Technol..

[109] Yao Z., Chong G., Guo H. (2024). Deep Eutectic Solvent Pretreatment and Green Separation of Lignocellulose. Appl. Sci..

[110] Hong S., Shen X.-J., Xue Z., Sun Z., Yuan T.-Q. (2020). Structure–function relationships of deep eutectic solvents for lignin extraction and chemical transformation. Green Chem..

[111] Meraj A., Jawaid M., Singh S.P., Nasef M.M., Ariffin H., Fouad H., Abu-Jdayil B. (2024). Isolation and characterisation of lignin using natural deep eutectic solvents pretreated kenaf fibre biomass. Sci. Rep..

[112] Li P., Zhang Z., Zhang X., Li K., Jin Y., Wu W. (2023). DES: Their effect on lignin and recycling performance. RSC Adv..

[113] Lin K.-T., Wang C., Guo M.F., Aprà E., Ma R., Ragauskas A.J., Zhang X. (2023). Lignin with controlled structural properties by N-heterocycle-based deep eutectic solvent extraction. Proc. Natl. Acad. Sci. USA.

[114] Hu M., Yu Y., Li X., Wang X., Liu Y. (2023). The dawn of aqueous deep eutectic solvents for lignin extraction. Green Chem..

[115] Ghareh Bagh F.S., Ray S., Seth R. (2021). Optimizing lignin extraction from Kraft black liquor using protic Ionic liquids. Biomass Bioenergy.

[116] Younesi-Kordkheili H., Pizzi A. (2020). Ionic liquid-modified lignin as a bio-coupling agent for natural fiber-recycled polypropylene composites. Compos. Part B Eng..

[117] Liao J.J., Abd Latif N.H., Trache D., Brosse N., Hussin M.H. (2020). Current advancement on the isolation, characterization and application of lignin. Int. J. Biol. Macromol..

[118] Achinivu E.C. (2018). Protic ionic liquids for lignin extraction—A lignin characterization study. Int. J. Mol. Sci..

[119] Usmani Z., Sharma M., Gupta P., Karpichev Y., Gathergood N., Bhat R., Gupta V.K. (2020). Ionic liquid based pretreatment of lignocellulosic biomass for enhanced bioconversion. Bioresour. Technol..

[120] Kang S., Li X., Fan J., Chang J. (2012). Characterization of Hydrochars Produced by Hydrothermal Carbonization of Lignin, Cellulose, d-Xylose, and Wood Meal. Ind. Eng. Chem. Res..

[121] Djandja O.S., Liew R.K., Liu C., Liang J., Yuan H., He W., Feng Y., Lougou B.G., Duan P.-G., Lu X. (2023). Catalytic hydrothermal carbonization of wet organic solid waste: A review. Sci. Total Environ..

[122] Rodríguez Correa C., Stollovsky M., Hehr T., Rauscher Y., Rolli B., Kruse A. (2017). Influence of the Carbonization Process on Activated Carbon Properties from Lignin and Lignin-Rich Biomasses. ACS Sustain. Chem. Eng..

[123] Sadish O., Paul Sebastian S., Banu K.S.P., Mahendran R. (2019). Hydrochar as an Energy Alternative to Coal: Effect of Temperature on Hydrothermal Carbonization of Paper Board Mill Sludge. Int. J. Curr. Microbiol. Appl. Sci..

[124] Román S., Libra J., Berge N., Sabio E., Ro K., Li L., Ledesma B., Álvarez A., Bae S. (2018). Hydrothermal Carbonization: Modeling, Final Properties Design and Applications: A Review. Energies.

[125] He C., Giannis A., Wang J.-Y. (2013). Conversion of sewage sludge to clean solid fuel using hydrothermal carbonization: Hydrochar fuel characteristics and combustion behavior. Appl. Energy.

[126] Wu S., Wang Q., Fang M., Wu D., Cui D., Pan S., Bai J., Xu F., Wang Z. (2023). Hydrothermal carbonization of food waste for sustainable biofuel production: Advancements, challenges, and future prospects. Sci. Total Environ..

[127] Le H.S., Chen W.-H., Ahmed S.F., Said Z., Rafa N., Le A.T., Ağbulut Ü., Veza I., Nguyen X.P., Duong X.Q. (2022). Hydrothermal carbonization of food waste as sustainable energy conversion path. Bioresour. Technol..

[128] Wang T., Zhai Y., Zhu Y., Li C., Zeng G. (2018). A review of the hydrothermal carbonization of biomass waste for hydrochar formation: Process conditions, fundamentals, and physicochemical properties. Renew. Sustain. Energy Rev..

[129] Kim D., Cheon J., Kim J., Hwang D., Hong I., Kwon O.H., Park W.H., Cho D. (2017). Extraction and characterization of lignin from black liquor and preparation of biomass-based activated carbon there-from. Carbon Lett..

[130] He C., Huang M., Zhao L., Lei Y., He J., Tian D., Zeng Y., Shen F., Zou J. (2022). Enhanced electrochemical performance of porous carbon from wheat straw as remolded by hydrothermal processing. Sci. Total Environ..

[131] Sun G., Qiu L., Zhu M., Kang K., Guo X. (2018). Activated carbons prepared by hydrothermal pretreatment and chemical activation of Eucommia ulmoides wood for supercapacitors application. Ind. Crops Prod..

[132] Al-Kaabi Z., Pradhan R., Thevathasan N., Gordon A., Chiang Y.W., Dutta A. (2019). Bio-carbon production by oxidation and hydrothermal carbonization of paper recycling black liquor. J. Clean. Prod..

[133] Latham K.G., Matsakas L., Figueira J., Rova U., Christakopoulos P., Jansson S. (2021). Examination of how variations in lignin properties from Kraft and organosolv extraction influence the physicochemical characteristics of hydrothermal carbon. J. Anal. Appl. Pyrolysis.

[134] Khandelwal K., Seraj S., Nanda S., Azargohar R., Dalai A.K. (2024). Subcritical water conversion of biomass to biofuels, chemicals and materials: A review. Environ. Chem. Lett..

[135] Yabalak E., Akay S., Kayan B., Gizir A.M., Yang Y. (2023). Solubility and decomposition of organic compounds in subcritical water. Molecules.

[136] Boucard H., Weiss-Hortala E., Gueye F., Espitalier F., Barna R. (2020). Insights in mechanisms of carbonaceous microparticles formation from black liquor hydrothermal conversion. J. Supercrit. Fluids.

[137] Nizamuddin S., Baloch H.A., Griffin G.J., Mubarak N.M., Bhutto A.W., Abro R., Mazari S.A., Ali B.S. (2017). An overview of effect of process parameters on hydrothermal carbonization of biomass. Renew. Sustain. Energy Rev..

[138] Curmi H., Chirat C., Roubaud A., Peyrot M., Haarlemmer G., Lachenal D. (2022). Extraction of phenolic compounds from sulfur-free black liquor thanks to hydrothermal treatment before the production of syngas for biofuels. J. Supercrit. Fluids.

[139] Lynam J.G., Reza M.T., Yan W., Vásquez V.R., Coronella C.J. (2014). Hydrothermal carbonization of various lignocellulosic biomass. Biomass Convers. Biorefinery.

[140] Koprivica M., Petrović J., Ercegović M., Simić M., Milojković J., Šoštarić T., Dimitrijević J. (2024). Improvement of combustible characteristics of Paulownia leaves via hydrothermal carbonization. Biomass Convers. Biorefinery.

[141] Musa U., Castro-Díaz M., Uguna C.N., Snape C.E. (2022). Effect of process variables on producing biocoals by hydrothermal carbonisation of pine Kraft lignin at low temperatures. Fuel.

[142] Latham K.G., Matsakas L., Figueira J., Kozyatnyk I., Rova U., Christakopoulos P., Jansson S. (2022). Impact of temperature and residence time on the hydrothermal carbonization of organosolv lignin. J. Anal. Appl. Pyrolysis.

[143] Salcedo-Puerto O., Mendoza-Martinez C., Saari J., Vakkilainen E. (2024). Hydrothermal carbonization of industrial kraft lignin: Assessment of operational parameters. Fuel.

[144] Lachos-Perez D., Torres-Mayanga P.C., Abaide E.R., Zabot G.L., De Castilhos F. (2022). Hydrothermal carbonization and Liquefaction: Differences, progress, challenges, and opportunities. Bioresour. Technol..

[145] Sharma T., Hakeem I.G., Gupta A.B., Joshi J., Shah K., Vuppaladadiyam A.K., Sharma A. (2024). Parametric influence of process conditions on thermochemical techniques for biochar production: A state-of-the-art review. J. Energy Inst..

[146] Kang S., Li X., Fan J., Chang J. (2012). Solid fuel production by hydrothermal carbonization of black liquor. Bioresour. Technol..

[147] Wikberg H., Ohra-aho T., Pileidis F., Titirici M.-M. (2015). Structural and morphological changes in kraft lignin during hydrothermal carbonization. ACS Sustain. Chem. Eng..

[148] Evcil T., Simsir H., Ucar S., Tekin K., Karagoz S. (2020). Hydrothermal carbonization of lignocellulosic biomass and effects of combined Lewis and Brønsted acid catalysts. Fuel.

[149] Rasaq W.A., Okpala C.O.R., Igwegbe C.A., Białowiec A. (2024). Catalyst-Enhancing Hydrothermal Carbonization of Biomass for Hydrochar and Liquid Fuel Production—A Review. Materials.

[150] Le T.-H., Wang S., Kim B.-S., Nam H., Lee D. (2024). Advancements and challenges in catalytic hydrothermal liquefaction of lignocellulosic biomass: A comprehensive review. Chem. Eng. J..

[151] Liu X., Lu J., Fu M., Zheng H., Chen Q. (2022). Activated carbon induced hydrothermal carbonization for the treatment of cotton pulp black liquor. J. Water Process Eng..

[152] Muddasar M., Mushtaq M., Beaucamp A., Kennedy T., Culebras M., Collins M.N. (2024). Synthesis of sustainable lignin precursors for hierarchical porous carbons and their efficient performance in energy storage applications. ACS Sustain. Chem. Eng..

[153] Tong Y., Yang J., Li J., Cong Z., Wei L., Liu M., Zhai S., Wang K., An Q. (2023). Lignin-derived electrode materials for supercapacitor applications: Progress and perspectives. J. Mater. Chem. A.

[154] Heidarinejad Z., Dehghani M.H., Heidari M., Javedan G., Ali I., Sillanpää M. (2020). Methods for preparation and activation of activated carbon: A review. Environ. Chem. Lett..

[155] Jain A., Jayaraman S., Balasubramanian R., Srinivasan M. (2014). Hydrothermal pre-treatment for mesoporous carbon synthesis: Enhancement of chemical activation. J. Mater. Chem. A.

[156] Plavniece A., Dobele G., Volperts A., Zhurinsh A. (2022). Hydrothermal carbonization vs. pyrolysis: Effect on the porosity of the activated carbon materials. Sustainability.

[157] Wang H., Feng P., Fu F., Yu X., Yang D., Zhang W., Niu L., Qiu X. (2022). Lignin-derived carbon materials for catalysis and electrochemical energy storage. Carbon Neutralization.

[158] Pan Z., Yu S., Wang L., Li C., Meng F., Wang N., Zhou S., Xiong Y., Wang Z., Wu Y. (2023). Recent advances in porous carbon materials as electrodes for supercapacitors. Nanomaterials.

[159] Wang C., Yan B., Zheng J., Feng L., Chen Z., Zhang Q., Liao T., Chen J., Jiang S., Du C. (2022). Recent progress in template-assisted synthesis of porous carbons for supercapacitors. Adv. Powder Mater..

[160] Li H., Zhao Y., Liu S., Li P., Yuan D., He C. (2020). Hierarchical porous carbon monolith derived from lignin for high areal capacitance supercapacitors. Microporous Mesoporous Mater..

[161] Song Y., Liu J., Sun K., Xu W. (2017). Synthesis of sustainable lignin-derived mesoporous carbon for supercapacitors using a nano-sized MgO template coupled with Pluronic F127. RSC Adv..

[162] Yin J., Zhang W., Alhebshi N.A., Salah N., Alshareef H.N. (2020). Synthesis strategies of porous carbon for supercapacitor applications. Small Methods.

[163] Zhang M., He L., Shi T., Zha R. (2018). Nanocasting and direct synthesis strategies for mesoporous carbons as supercapacitor electrodes. Chem. Mater..

[164] Zhang W., Lin H., Lin Z., Yin J., Lu H., Liu D., Zhao M. (2015). 3D hierarchical porous carbon for supercapacitors prepared from lignin through a facile template-free method. ChemSusChem.

[165] Li H., Shi F., An Q., Zhai S., Wang K., Tong Y. (2021). Three-dimensional hierarchical porous carbon derived from lignin for supercapacitors: Insight into the hydrothermal carbonization and activation. Int. J. Biol. Macromol..

[166] Poudel M.B., Kim H.J. (2022). Confinement of Zn-Mg-Al-layered double hydroxide and α-Fe_2_O_3_ nanorods on hollow porous carbon nanofibers: A free-standing electrode for solid-state symmetric supercapacitors. Chem. Eng. J..

[167] Wang H., Niu H., Wang H., Wang W., Jin X., Wang H., Zhou H., Lin T. (2021). Micro-meso porous structured carbon nanofibers with ultra-high surface area and large supercapacitor electrode capacitance. J. Power Sources.

[168] Muddasar M., Culebras M., Collins M.N. (2024). Lignin and its carbon derivatives: Synthesis techniques and their energy storage applications. Mater. Today Sustain..

[169] Liu F., Wang Q., Zhai G., Xiang H., Zhou J., Jia C., Zhu L., Wu Q., Zhu M.J.N.C. (2022). Continuously processing waste lignin into high-value carbon nanotube fibers. Nat. Commun..

[170] Singh M., Gupta A., Sundriyal S., Jain K., Dhakate S. (2021). Kraft lignin-derived free-standing carbon nanofibers mat for high-performance all-solid-state supercapacitor. Mater. Chem. Phys..

[171] Zhuang X., Zhan H., Song Y., He C., Huang Y., Yin X., Wu C. (2019). Insights into the evolution of chemical structures in lignocellulose and non-lignocellulose biowastes during hydrothermal carbonization (HTC). Fuel.

[172] Cai J., Naraghi M. (2018). Non-intertwined graphitic domains leads to super strong and tough continuous 1D nanostructures. Carbon.

[173] Shi F., Zhao S., Yang J., Tong Y., Li J., Zhai S., Zhao X., Wu S., Li H., An Q. (2022). Nickel–cobalt bimetallic tungstate decorated 3D hierarchical porous carbon derived from lignin for high-performance supercapacitor applications. J. Mater. Chem. A.

[174] Fu F., Yang D., Wang H., Qian Y., Yuan F., Zhong J., Qiu X. (2019). Three-dimensional porous framework lignin-derived carbon/ZnO composite fabricated by a facile electrostatic self-assembly showing good stability for high-performance supercapacitors. ACS Sustain. Chem. Eng..

[175] Chaleawlert-Umpon S., Berthold T., Wang X., Antonietti M., Liedel C. (2017). Kraft lignin as electrode material for sustainable electrochemical energy storage. Adv. Mater. Interfaces.

[176] Yang W., Qu Y., Zhou B., Li C., Jiao L., Dai H. (2021). Value-added utilization of lignin-derived aromatic oligomers as renewable charge-storage materials. Ind. Crops Prod..

[177] Shi F., Li J., Xiao J., Zhao X., Li H., An Q., Zhai S., Wang K., Wei L., Tong Y. (2021). Three-dimensional hierarchical porous lignin-derived carbon/WO3 for high-performance solid-state planar micro-supercapacitor. Int. J. Biol. Macromol..

[178] Chen X., Liu C., Fang Y., Ai X., Zhong F., Yang H., Cao Y. (2022). Understanding of the sodium storage mechanism in hard carbon anodes. Carbon Energy.

[179] Meng Q., Chen B., Jian W., Zhang X., Sun S., Wang T., Zhang W. (2023). Hard carbon anodes for sodium-ion batteries: Dependence of the microstructure and performance on the molecular structure of lignin. J. Power Sources.

[180] Beaucamp A., Muddasar M., Amiinu I.S., Leite M.M., Culebras M., Latha K., Gutiérrez M.C., Rodriguez-Padron D., del Monte F., Kennedy T. (2022). Lignin for energy applications–state of the art, life cycle, technoeconomic analysis and future trends. Green Chem..

[181] Chen M., Luo F., Liao Y., Liu C., Xu D., Wang Z., Liu Q., Wang D., Ye Y., Li S. (2022). Hard carbon derived for lignin with robust and low-potential sodium ion storage. J. Electroanal. Chem..

[182] Yadlapalli R.T., Alla R.R., Kandipati R., Kotapati A. (2022). Super capacitors for energy storage: Progress, applications and challenges. J. Energy Storage.

[183] Yang F., Zhang F., Song Y., Wang X., Yang Y. (2024). Hierarchical porous hollow carbon microsphere/carbon nanotube heterostructures for high-performance supercapacitor. J. Energy Storage.

[184] Zhang S., Zhang Q., Ma R., Feng X., Chen F., Wang D., Zhang B., Wang Y., Guo N., Xu M. (2024). Boosting the capacitive performance by constructing O, N co-doped hierarchical porous structure in carbon for supercapacitor. J. Energy Storage.

[185] Wan X., Shen F., Hu J., Huang M., Zhao L., Zeng Y., Tian D., Yang G., Zhang Y. (2021). 3-D hierarchical porous carbon from oxidized lignin by one-step activation for high-performance supercapacitor. Int. J. Biol. Macromol..

[186] Sun Y., Xu D., Wang S. (2022). Self-assembly of biomass derivatives into multiple heteroatom-doped 3D-interconnected porous carbon for advanced supercapacitors. Carbon.

[187] Feng X., Bai Y., Liu M., Li Y., Yang H., Wang X., Wu C. (2021). Untangling the respective effects of heteroatom-doped carbon materials in batteries, supercapacitors and the ORR to design high performance materials. Energy Environ. Sci..

[188] Abbas Q., Raza R., Shabbir I., Olabi A. (2019). Heteroatom doped high porosity carbon nanomaterials as electrodes for energy storage in electrochemical capacitors: A review. J. Sci. Adv. Mater. Devices.

[189] Luo L., Zhou Y., Yan W., Wu X., Wang S., Zhao W. (2020). Two-step synthesis of B and N co-doped porous carbon composites by microwave-assisted hydrothermal and pyrolysis process for supercapacitor application. Electrochim. Acta.

[190] Li W., Zhang W., Xu Y., Wang G., Sui W., Xu T., Yuan Z., Si C. (2024). Heteroatom-doped lignin derived carbon materials for improved electrochemical performance: Synthesis, mechanism, and applications in advanced supercapacitors. Chem. Eng. J..

[191] Wang S., Feng J., Pan H. (2022). Facile preparation of nitrogen-doped hierarchical porous carbon derived from lignin with KCl for supercapacitors. Colloids Surf. A Physicochem. Eng. Asp..

[192] Zhai S., Li K., Li C., Zhai C., Han Q., Zhang Z., Fu Y., Li X., Jin K., Cai Z. (2024). Lignin-derived N, S-codoped hierarchical porous carbons with high mesoporous rate for sustainable supercapacitive energy storage. J. Energy Storage.

[193] Li S., Luo X., Xiao H., Li D., Chen Y. (2023). Nitrogen and sulfur codoped hierarchical porous carbon derived from lignin for high-performance zinc ion capacitors. ACS Appl. Energy Mater..

[194] Wanison R., Syahputra W.N.H., Kammuang-lue N., Sakulchangsatjatai P., Chaichana C., Shankar V.U., Suttakul P., Mona Y. (2024). Engineering aspects of sodium-ion battery: An alternative energy device for Lithium-ion batteries. J. Energy Storage.

[195] Kim T., Song W., Son D.-Y., Ono L.K., Qi Y. (2019). Lithium-ion batteries: Outlook on present, future, and hybridized technologies. J. Mater. Chem. A.

[196] Xi Y., Wang Y., Yang D., Liu W., Li Q., Qiu X. (2019). K_2_CO_3_ activation enhancing the graphitization of porous lignin carbon derived from enzymatic hydrolysis lignin for high performance lithium-ion storage. J. Alloys Compd..

[197] Zhao L., Ding B., Qin X.Y., Wang Z., Lv W., He Y.B., Yang Q.H., Kang F. (2022). Revisiting the roles of natural graphite in ongoing lithium-ion batteries. Adv. Mater..

[198] Zhao W., Zhao C., Wu H., Li L., Zhang C. (2024). Progress, challenge and perspective of graphite-based anode materials for lithium batteries: A review. J. Energy Storage.

[199] Aslam M.K., Niu Y., Hussain T., Tabassum H., Tang W., Xu M., Ahuja R. (2021). How to avoid dendrite formation in metal batteries: Innovative strategies for dendrite suppression. Nano Energy.

[200] Sabaghi D., Wang G., Mikhailova D., Morag A., Omar A., Li D., Vand S.K.H., Yu M., Feng X., Nia A.S. (2023). High energy density and durable pouch-cell graphite-based dual ion battery using concentrated hybrid electrolytes. J. Power Sources.

[201] Liu S., Gu B., Chen Z., Zhan R., Wang X., Feng R., Sun Y. (2024). Suppressing dendritic metallic Li formation on graphite anode under battery fast charging. J. Energy Chem..

[202] Jia G., Yu Y., Wang X., Jia C., Hu Z., Yu S., Xiang H., Zhu M. (2023). Highly conductive and porous lignin-derived carbon fibers. Mater. Horiz..

[203] Huang S., Yang D., Zhang W., Qiu X., Li Q., Li C. (2021). Dual-templated synthesis of mesoporous lignin-derived honeycomb-like porous carbon/SiO2 composites for high-performance Li-ion battery. Microporous Mesoporous Mater..

[204] Culebras M., Geaney H., Beaucamp A., Upadhyaya P., Dalton E., Ryan K.M., Collins M.N. (2019). Bio-derived carbon nanofibres from lignin as high-performance Li-ion anode materials. ChemSusChem.

[205] Du Y.-F., Sun G.-H., Li Y., Cheng J.-Y., Chen J.-P., Song G., Kong Q.-Q., Xie L.-J., Chen C.-M. (2021). Pre-oxidation of lignin precursors for hard carbon anode with boosted lithium-ion storage capacity. Carbon.

[206] Jiang Q., Ni Y., Zhang Q., Gao J., Wang Z., Yin H., Jing Y., Wang J. (2022). Sustainable nitrogen self-doped carbon nanofibers from biomass chitin as anodes for high-performance lithium-ion batteries. Energy Fuels.

[207] Liu J., Mei X.-W., Peng F. (2023). Lignin derived porous carbon with favorable mesoporous contributions for highly efficient ionic liquid-based supercapacitors. Chin. Chem. Lett..

[208] Nieto N., Porte J., Saurel D., Djuandhi L., Sharma N., Lopez-Urionabarrenechea A., Palomares V., Rojo T. (2023). Use of hydrothermal carbonization to improve the performance of biowaste-derived hard carbons in sodium ion-batteries. ChemSusChem.

[209] Wang H., Niu H., Shu K., Sun L., Wang Y., Du Y., Han Y., Yang C., Kang Y.-M. (2025). Regulating the “core-shell” microstructure of hard carbon through sodium hydroxide activation for achieving high-capacity SIBs anode. J. Mater. Sci. Technol..

[210] Tang Z., Liu R., Jiang D., Cai S., Li H., Sun D., Tang Y., Wang H. (2024). Regulating the pore structure of biomass-derived hard carbon for an advanced sodium-ion battery. ACS Appl. Mater. Interfaces.

[211] Song Z., Di M., Chen S., Bai Y. (2023). Three-dimensional N/O co-doped hard carbon anode enabled superior stabilities for sodium-ion batteries. Chem. Eng. J..

[212] Muruganantham R., Wang F.-M., Liu W.-R. (2022). A green route N, S-doped hard carbon derived from fruit-peel biomass waste as an anode material for rechargeable sodium-ion storage applications. Electrochim. Acta.

[213] Tao S., Xu W., Zheng J., Kong F., Cui P., Wu D., Qian B., Chen S., Song L. (2021). Soybean roots-derived N, P Co-doped mesoporous hard carbon for boosting sodium and potassium-ion batteries. Carbon.

[214] Alvin S., Chandra C., Kim J. (2020). Extended plateau capacity of phosphorus-doped hard carbon used as an anode in Na-and K-ion batteries. Chem. Eng. J..

[215] Lin X., Liu Y., Tan H., Zhang B. (2020). Advanced lignin-derived hard carbon for Na-ion batteries and a comparison with Li and K ion storage. Carbon.

